# An antibody that neutralizes SARS-CoV-1 and SARS-CoV-2 by binding to a conserved spike epitope outside the receptor binding motif

**DOI:** 10.1126/sciimmunol.abp9962

**Published:** 2022-08-04

**Authors:** Yan Fang, Pengcheng Sun, Xuping Xie, Mingjian Du, Fenghe Du, Jianfeng Ye, Birte K. Kalveram, Jessica A. Plante, Kenneth S. Plante, Bo Li, Xiao-chen Bai, Pei-Yong Shi, Zhijian J. Chen

**Affiliations:** ^1^ Department of Molecular Biology, University of Texas Southwestern Medical Center, Dallas, TX 75390-9148, USA; ^2^ Department of Biochemistry and Molecular Biology, University of Texas Medical Branch, 301 University Boulevard, Galveston, TX 77555-0144, USA; ^3^ Lyda Hill Department of Bioinformatics, University of Texas Southwestern Medical Center, Dallas, TX 75390, USA; ^4^ Department of Biophysics, University of Texas Southwestern Medical Center, Dallas, TX 75390, USA; ^5^ Department of Microbiology and Immunology, University of Texas Medical Branch, Galveston TX, USA; ^6^ Institute for Human Infections and Immunity, University of Texas Medical Branch, Galveston, TX, USA; ^7^ World Reference Center for Emerging Viruses and Arboviruses, University of Texas Medical Branch, Galveston, TX, USA; ^8^ Sealy Institute for Drug Discovery, University of Texas Medical Branch, Galveston, TX 77555, USA; ^9^ Center for Inflammation Research, University of Texas Southwestern Medical Center, Dallas, TX 75390-9148, USA; ^10^ Howard Hughes Medical Institute, University of Texas Southwestern Medical Center, Dallas, TX 75390-9148, USA

## Abstract

The rapid evolution of SARS-CoV-2 viruses, such as the Omicron variants which are highly transmissible and immune evasive, underscores the need to develop therapeutic antibodies with broad neutralizing activities. Here, we used the LIBRA-seq technology, which identified SARS-CoV-2 specific B cells via DNA-barcoding and subsequently single cell sequenced BCRs, to identify an antibody, SW186, which could neutralize major SARS-CoV-2 variants of concern, including Beta, Delta, and Omicron, as well as SARS-CoV-1. The cryo-EM structure of SW186 bound to the receptor-binding domain (RBD) of the viral spike protein showed that SW186 interacted with an epitope of the RBD that is not at the interface of its binding to the ACE2 receptor but highly conserved among SARS coronaviruses. This epitope encompasses a glycosylation site (N343) of the viral spike protein. Administration of SW186 in mice after they were infected with SARS-CoV-2 Alpha, Beta, or Delta variants reduced the viral loads in the lung. These results demonstrated that SW186 neutralizes diverse SARS coronaviruses by binding to a conserved RBD epitope, which could serve as a target for further antibody development.

## Teaser

A SARS-CoV-2 antibody with broad neutralizing activity against SARS-CoV-1 and SARS-CoV-2.

## Introduction

SARS-CoV-2 has caused one of the worst pandemics in human history (*1*). The virus is airborne and enters cells in the airway through the interaction between the receptor binding domain (RBD) of the viral spike (S) protein and angiotensin-converting enzyme 2 (ACE2) on the host cell membrane (*2*). SARS-CoV-2 neutralizing monoclonal antibodies (mAbs), together with vaccines and antiviral drugs, are important tools to control the COVID-19 pandemic. Although many mAbs have been developed, most were developed for the early SARS-CoV-2 strains (*3*). The SARS-CoV-2 variants, including Alpha (B.1.1.7), Beta (B.1.351), Delta (B.1.617.2), Gamma (P.1), Lambda (C.37), Mu (B.1.621), and Omicron (B.1.1.529 and its sublineages, including BA.1, BA.2, BA.2.12.1, BA.3, BA.4 and BA.5), developed resistance to therapeutic mAbs to varying extents (*3*–*6*). Significantly, the recently emerged Omicron variants harbor more than 15 mutations in the RBD of the spike protein and exhibit strong resistance to most therapeutic mAbs and even the plasma of vaccinated individuals (*5*, *6*). Most therapeutic antibodies were developed based on their ability to compete with the binding of ACE2 to the spike protein's receptor-binding motif (RBM). This strategy allows for fast antibody development, but most if not all therapeutic mAbs directed against the RBM lost their activities against the Omicron variant (*7*). Therefore, there is an urgent need to develop new broadly neutralizing antibodies against current and future coronaviruses.

Here, we isolated a panel of SARS-CoV-2 antibodies from mice immunized with the viral spike protein and identified that SW186 monoclonal antibody as showing the best neutralizing activity against all variants tested, including the Omicron variants, and SARS-CoV-1. Using cryo-electron microscopy (cryo-EM) reconstruction, we determined the structure of the fragment antigen-binding region (Fab) of SW186 bound to the outer surface of RBD, which is distinct from the RBM that binds to ACE2. The SW186 antibody reduced SARS-CoV-2 viral loads in the lungs of mice, demonstrating a therapeutic effect. Thus, SW186 is a broadly neutralizing antibody against SARS coronaviruses and binds to a unique and conserved epitope of the spike protein.

## RESULTS

### Identification of monoclonal antibodies against SARS-CoV-2 spike protein

**Fig. 1.**
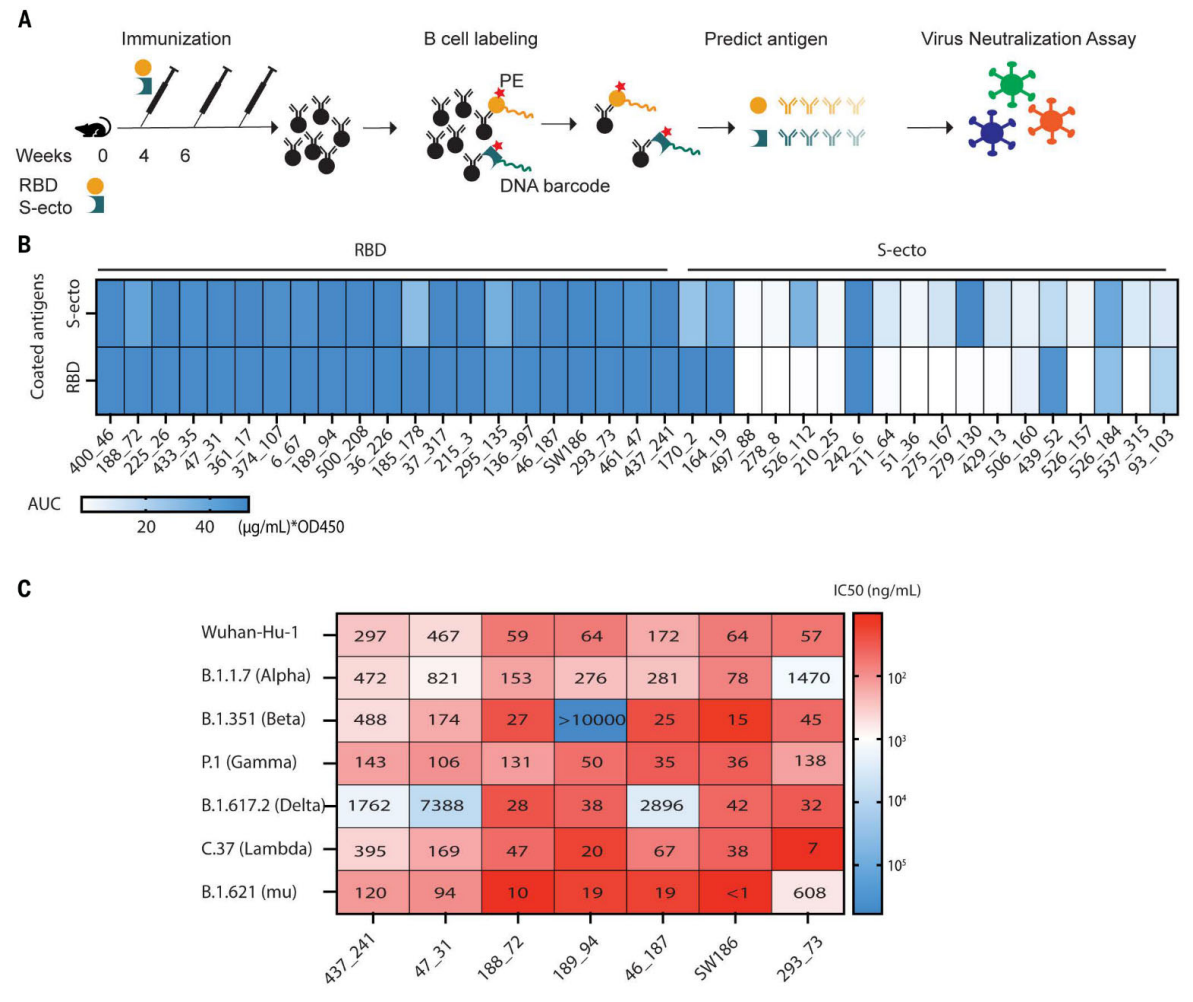
Identification of broadly neutralizing antibodies against SARS-CoV-2 variants. (A) A pipeline of antibody identification and characterization. Splenocytes from mice immunized with the RBD or the ectodomain of spike protein (S-ecto) were stained with biotinylated antigens that were labeled with DNA oligonucleotide barcodes. IgG+ antigen+ cells were sorted and processed for scRNA-seq to obtain the antigen-specific BCR sequences. Each antibody was analyzed by ELISA and neutralization assays using pseudoviruses displaying the spike proteins from SARS-CoV-2 variants. (B) Analyses of antibody binding to the spike protein and RBD. The heatmap shows the area under the curve (AUC) values measured by ELISA assay. The unit of AUC is (μg/mL) * OD450. The y-axis indicates the antigens used to coat the ELISA plates, and the labels on the top indicate the antigens used to immunize the mice. (C) IC_50_ values from the neutralization assays of seven selected antibodies using pseudotyped viruses displaying the spike proteins from indicated SARS-CoV-2 variants. The heatmap shows the -log(IC_50_), and the numbers in the plot represent the IC_50_ values in ng/mL (representative data from three independent experiments).

To obtain antibodies targeting the viral spike protein, we immunized C57BL/6J mice with the spike ectodomain (S-ecto) or RBD protein of the Wuhan-hu-1 strain, using the STING agonist 2’3′-cGAMP as an adjuvant (*8*). After boosting twice, splenic memory B cells displaying IgGs against S-ecto or RBD were isolated (Fig. 1A, Fig. S1). To reduce non-specific binding, we used LIBRA-seq (linking B cell receptor to antigen specificity through sequencing) (*9*). In brief, we stained splenic B cells with S-ecto or RBD protein that was biotinylated and labeled with DNA barcodes. Cells were then stained with PE-streptavidin, and the IgG+PE+ cells were sorted and processed for single-cell RNA sequencing (scRNA-seq). The DNA barcode on each B cell indicated its antigen specificity. To further reduce background noise, we selected B cells positive for both PE-streptavidin and DNA barcodes as antigen-specific B cells (Fig. S2A). This strategy removed clonally expanded B cells without DNA barcodes (Fig. S2C). In total, 533 B cells with paired heavy chains and light chains as well as DNA barcodes (UMI > 100) were isolated (Table S1). B cells from the same V-D-J lineage share similar CDR3 sequences and epitopes. Therefore, we selected BCRs with distinct V-D-J lineage and clonally expanded B cell clones (frequency ≥ 3) for further analyses to maximize the epitope diversity (Fig. S2A and Fig. S2B).

We inserted the DNA encoding the variable regions of identified BCRs into a human IgG1 antibody backbone to generate murine-human chimeric antibodies and tested their antigen specificity using ELISA. The results showed that all the antibodies from the B cells selected with the RBD barcode bound both S-ecto and RBD, whereas the antibodies from the B cells selected with the S-ecto barcode showed diverse binding abilities against these two antigens (Fig. 1B). In total, we obtained 27 antibodies targeting the RBD and 7 antibodies targeting the non-RBD region of the S-ecto protein (Table S1).

### Screening for neutralizing antibodies against multiple SARS-CoV-2 variants of concern

**Fig. 2.**
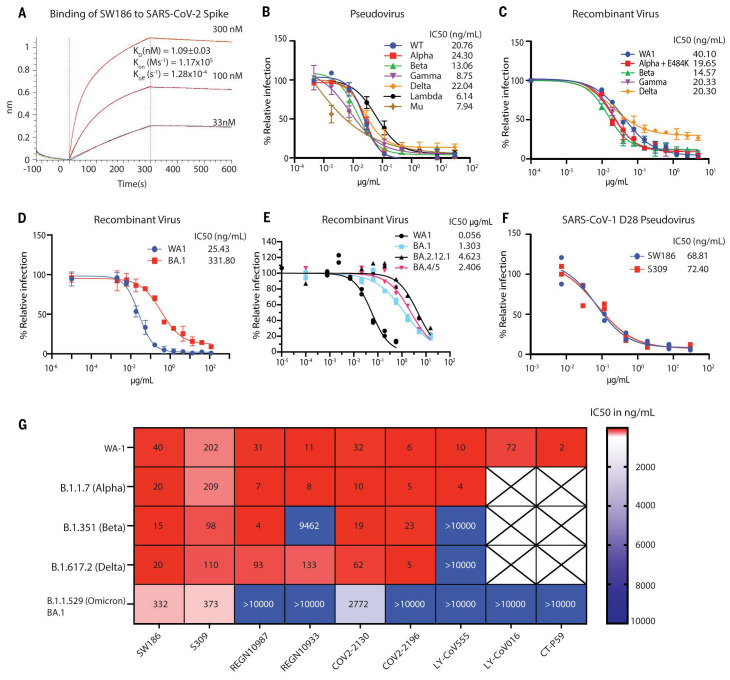
SW186 has broad neutralizing activity against SARS-CoV-2 variants of concern. (A) The kinetics of binding of SW186 to the spike protein using BioLayer Interferometry (BLI). The biotinylated S-ecto protein was loaded on the streptavidin sensor and then SW186 was applied at indicated concentrations. The K_D_ value is mean ± SD, n=3. (B) Neutralization assays were performed using pseudotyped viruses displaying the spike protein from indicated SARS-CoV-2 variants. The values are mean ± SD, n=3. (C-E) Neutralization assays were performed using recombinant infectious SARS-CoV-2 viruses as indicated. The values are mean ± SD, n=2. (F) Neutralization assays of SW186 and S309 against the SARS-CoV-1-D28 (deletion of C-terminal 28aa) pseudotyped virus. Each antibody dilution was done in duplicates. (G) The neutralization activities (IC_50_ at ng/mL) of monoclonal antibodies against SARS-CoV-2 variants were adapted from Fig. 2C-D and published studies (*4*–*6*). The heatmap shows the IC_50_ value.

To identify neutralizing antibodies against SARS-CoV-2 variants, we employed a virus neutralization assay using HIV-based pseudovirus displaying a SARS-CoV-2 spike protein (*10*). Seven antibodies exhibited neutralizing activities with IC_50_ < 100 ng/mL against pseudovirus displaying the spike protein from the original Wuhan-hu-1 strain of SARS-CoV2, including 437_241, 47_31, 188_72, 189_94, 46_187, SW186, 293_73 (Fig. S2D). We then generated pseudoviruses displaying the spike protein harboring mutations found in several variants, including Alpha (B.1.1.7), Beta (B.1.351), Delta (B.1.617.2), Gamma (P.1), Lambda (C.37), and Mu (B.1.621) (Fig. S3). Among the seven antibodies with high neutralizing activity against the Wuhan-hu-1 strain, SW186 showed the best neutralization activity against all variants tested (Fig. 1C, Fig. 2B). In addition to SW186, antibody 188_72 also showed broad neutralizing activity against different variants. Other antibodies, including 189_94, 46_187, 293_73, had neutralizing activity against most but not all variants tested (Fig. 1C).

Using BioLayer Interferometry (BLI), we determined the binding affinity of SW186 to spike-RBD with a K_d_ of ~ 1 nM (Fig. 2A). We further tested the neutralizing activity of SW186 against infectious mNeonGreen SARS-CoV-2 variants generated through reverse genetics (*11*). The antibody SW186 potently neutralized the WA1 strain of SARS-CoV-2 with an IC_50_ of ~40 ng/mL (Fig. 2C). It also potently neutralized multiple SARS-CoV-2 variants, including Alpha (B.1.1.7) that was engineered to carry an additional E484K mutation known to cause immune escape (*12*), Beta (B.1.351), Delta (B.1.617.2), and Gamma (P.1), with IC_50_s in the range of ~15-20 ng/mL (Fig. 2C). SW186 also neutralized the Omicron virus (B.1.529, BA.1) with an IC_50_ of 332 ng/mL (Fig. 2D), comparable to the published IC_50_ of S309, an antibody isolated from a patient infected with SARS-CoV-1 in 2003 (Fig. 2G) (*13*) (*4*–*6*). The Omicron virus has continued to mutate, generating more infectious and immune evasive variants, including BA.2, BA.4 and BA.5. We tested the ability of SW186 and S309 to neutralize recombinant SARS-CoV-2 viruses displaying the spike protein of BA.2.12.1 and BA.4/5 (BA.4 and B.A5 contain the same mutations in the spike protein). Both antibodies had a substantial decrease in their potency to neutralize these currently dominant Omicron variants (Fig. 2E and Fig. S10).

To determine if SW186 could neutralize a divergent coronavirus, such as SARS-CoV-1, we generated a pseudovirus displaying the spike protein of SARS-CoV-1. Both full-length spike protein and a protein lacking 28 amino acids at the C terminus were tested in this assay (*14*). Interestingly, SW186 neutralized the SARS-CoV-1 pseudoviruses with an IC50 of ~69 ng/mL, which was similar to that of S309 (Fig. 2F, Fig. S4A), suggesting that SW186 targets an epitope that was conserved between SARS-CoV-1 and SARS-CoV-2. However, S309 but not SW186 neutralized a pseudovirus displaying the spike protein of WIV1, a SARS-like coronavirus in bats (*15*) (Fig. S4B). Thus, SW186 and S309 targeted a similar but not identical epitope on the SARS coronaviruses.

### Structural analysis of SW186 Fab:Spike complex

To understand the mechanism of the broad neutralizing activity of SW186 against the SARS-CoV-2 variants, we determined the cryo-EM structure of a fragment antigen-binding region (Fab) of SW186 bound to the spike protein of the Wuhan-hu-1 strain SARS-CoV-2 (Fig. S5; Table S2). With local refinement, we obtained the cryo-EM structure of the SW186 Fab bound to RBD-subdomain 1 (SD1) of the spike protein at overall 3.4Å resolution, which allowed us to reveal the detailed binding interface (Fig. S5C and S5D). Only the variable region of the heavy chain (VH) and light chain (VL) of SW186 Fab could be built because of the flexibility. The spike protein exhibited an intermediate state between the ‘close’ and ‘open’ states, with the RBD slightly moving away from the top (Fig. S6). Surprisingly, even though the Fab of this antibody was in excess, the majority of the particles contained only one Fab bound to the partially opened RBD of one spike trimer in vitro, whereas only a small subset of particles contained two Fabs (Fig. S5D). These results raise the interesting possibility that the binding to one antibody might have the ability to lock the spike in a conformation that prevents it from binding to another antibody.

**Fig. 3.**
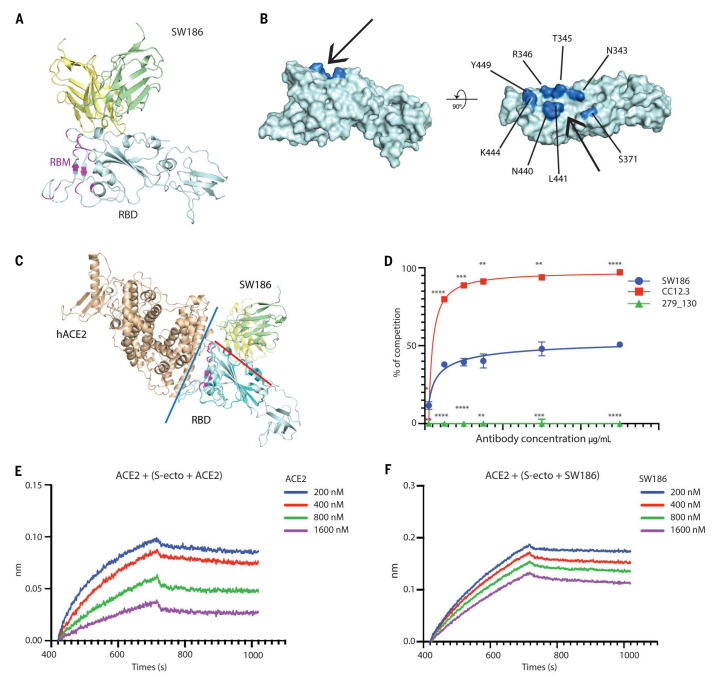
Structural analysis of SW186 Fab bound to RBD-SD1. (A) Overview of the locally refined structure of SW186 Fab bound to spike-RBD-SD1. Variable regions of the heavy chain (VH), light chain (VL), and RBD-SD1 are colored pale yellow, pale green, and pale cyan, respectively; receptor-binding motif (RBM) of the spike protein is colored magenta. (B) Surface presentation of RBD binding epitope of SW186. Critical residues on RBD are colored marine. The ‘minor groove’ is shown with an arrow. (C) Superposition of SW186 Fab bound to RBD-SD1 and hACE2 bound to RBD (PDB code: 6M17). hACE2 and RBD are colored wheat and cyan, respectively. Variable regions of the heavy chain (VH) and light chain (VL) are colored pale yellow and pale green, respectively. Red and blue lines show the interfaces between SW186 Fab and RBD and between hACE2 and RBD, respectively. The spike RBM is colored magenta. (D) Cell-based assay of the spike protein binding to ACE2 and competition by the antibody. Each data point represents triplicates. Two-way ANOVA was used to assess the significance of difference. * indicates statistical difference of the indicated antibody as compared to SW186. p-values: ≤ 0.05 (*), ≤ 0.01 (**), ≤ 0.001 (***), ≤ 0.0001 (****). (E-F) Kinetics of competitive binding of antibody SW186 and hACE2 to SARS-CoV-2 spike protein. For both panels, biotinylated hACE2-Fc was loaded onto the streptavidin sensor. S-ecto protein was pre-incubated with serially diluted ACE2-His_6_ (E; as a positive control) or SW186 (F) for 30 min at RT. The mixture was further loaded onto the hACE2-Fc coated sensor to detect the binding of S-ecto to ACE2.

Strikingly, SW186 recognized a region of the RBD located outside the RBM, which mediates binding to the receptor ACE2 (Fig. 3A). The epitope on the RBD was a minor groove comprising several conserved amino acids (Fig. 3B). Superimposing the structure of SW186 Fab bound to RBD with that of ACE2 bound to RBD revealed that the antibody did not bind at the interface between ACE2 and RBD (Fig. 3C). Cell-based competition experiments showed that SW186 partially competed with S-ecto in binding to ACE2 on the surface of Huh7 cells (Fig. 3D). The antibody CC12.3 (*16*), which binds the RBM of the spike protein, and 279_130, which binds to spike at a region outside the RBD (Fig. 1B), served as positive and negative controls, respectively (Fig. 3D). This partial competition of ACE2 binding to RBD by SW186 was confirmed by the BLI assay (Fig. 3E and 3F).

**Fig. 4.**
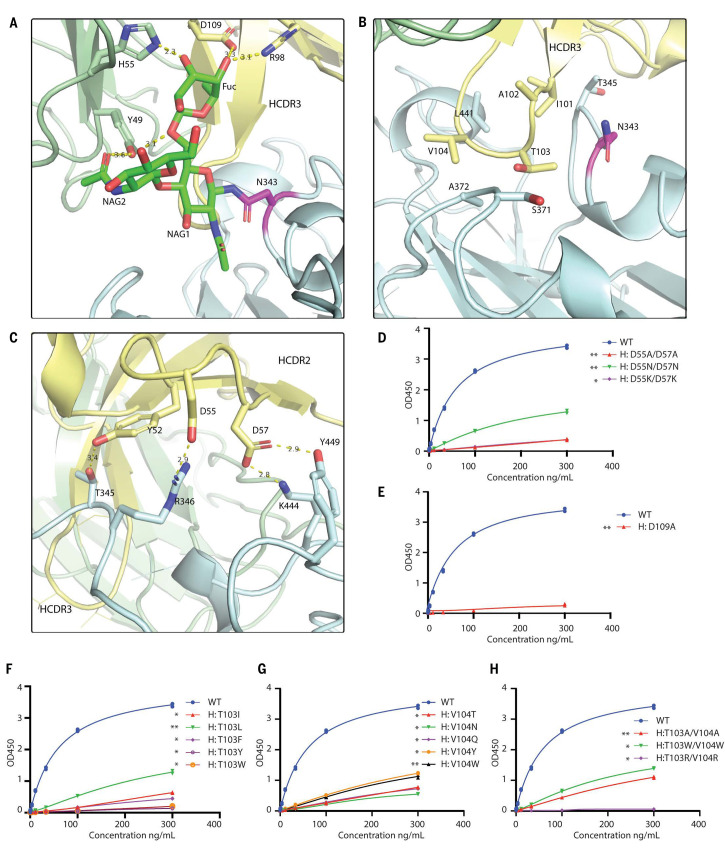
Structural analysis of SW186 Fab binding to RBD-SD1. (A) N343-linked glycan binds to SW186 Fab through extensive hydrogen bonding. NAG: N-Acetylglucosamine, Fuc: Fucose. N343-linked glycan is colored green, and N343 is colored magenta (B) CDR3 region of the heavy chain partially inserts into the minor groove on the side of RBD. (C) CDR2 region interacts with RBD by polar interactions. The residues involved in Fab-RBD binding are shown as sticks. Dash lines represent hydrogen bonds. (D-H) Mutational analysis of SW186 by ELISA. Indicated mutants of SW186 were serially diluted and tested for their binding with S-ecto protein that was coated on an ELISA plate. Experiments were performed in duplicate. All data points are shown in plots. * indicates the significant difference between the indicated SW186 mutants and WT SW186 at 300 ng/mL as calculated by two-way ANOVA. p-values: ≤ 0.05 (*), ≤ 0.01 (**), ≤ 0.001 (***), ≤ 0.0001 (****).

The interface between the SW186 antibody and RBD could be divided into three parts, each with its distinct features. First, SW186 interacted with N343-linked glycan on RBD via both heavy and light chains (Fig. 4A). Due to the flexibility of the polysaccharide chain, only three sugar molecules were modeled. These sugars formed extensive hydrogen bonds with R98 and D109 of the heavy chain and Y49 and H55 of the light chain. The N343-linked glycan on RBD functioned as a hook to pull the antibody toward the RBD. The ELISA-based binding assay showed that the D109A mutation on the heavy chain largely abolished the binding of the antibody with the spike protein (Fig. 4E, Fig. S7). These results suggested that SW186 bound to the epitope containing N343-glycan, which is important for virus entry into host cells by controlling the transition of the RBD from a glycan-shielded ‘down’ state to an open ‘up’ state (*17*). The importance of N343 glycosylation may explain why this residue is invariant among all SARS-CoV-2 variants and is also conserved in SARS-CoV-1(N330) (*18*).

Secondly, the SW186 heavy chain CDR3 (HCDR3) loop partially inserted into a ‘minor groove’ on the side of RBD, where I101, A102, T103, and V104 were mainly surrounded by the backbone of the RBD polypeptide chain (Fig. 3B, Fig. 4B). Mutations of T103 and V104, alone or in combination, to various amino acids impaired the ability of the antibody to bind the spike protein (Fig. 4F-4H). On the RBD side, N343, T345, S371, A372, and L441 were involved in the groove formation and interacted with HCDR3 through hydrophobic interactions. Because these interactions were mainly contributed by the polypeptide backbone rather than the side chains of the RBD minor groove, the binding of SW186 could be less affected by mutations in the RBD.

Thirdly, HCDR2 formed polar interactions with RBD via Y52, D55, and D57 (Fig. 4C). Y52 and D57 interacted with T345 and Y449 of RBD to form hydrogen bonds, while D55 and D57 formed salt bridges with R346 and K444 of RBD. Consistent with this model, substitutions of both D55 and D57 with Ala or Asn in the antibody drastically reduced its binding to the spike protein, whereas the mutation to Lys abolished the binding (Fig. 4D). Notably, R346K is a mutation found in a subset of the Omicron variant (i.e., BA.1.1) and was found to cause immune escape to most antibodies tested (*6*). However, SW186 effectively neutralized the Mu variant (B1.621), which harbors the R346K mutation, suggesting that this conservative substitution was tolerated by SW186 (Fig. 1C).

The Omicron spike proteins contain 15 (BA.1) or 17 (BA.2, BA.4, and BA.5) mutations from the original Wuhan-hu-1 strain. We mapped the mutations on the structure of RBD bound to SW186 and found that most mutations did not overlap with the epitope recognized by SW186 (Fig. S8A, Fig. S8I). Only two mutations, S371L (in BA.2) or S371F (in BA.2 or BA.4/5) and N440K, overlapped with the SW186 epitope, which could explain the reduced but still effective neutralization of the Omicron virus by SW186 (Fig. 2D and Fig. S8I). Except for S309 (*13*), most FDA-approved antibodies, including LY-COV016 (*19*), LY-COV055 (*20*), CT-P59 (*21*), REGN10933 + REGN10987 (*22*), AZD7442 (*10*), and ABBV-2B04 + ABBV-47D11 (*23*) bind to their epitopes on the RBD at the interface with ACE2 (Fig. S8C-I). The amino acids comprising these epitopes are extensively mutated in the Omicron (e.g., E484A and Q493R), explaining why these antibodies fail to neutralize the Omicron variant. S309 bound to an epitope that partially overlaps with that of SW186, including the N343-glycan (Fig. S8B and Fig. S9).

### SW186 protects mice against several SARS-CoV-2 variants of concerns

**Fig. 5.**
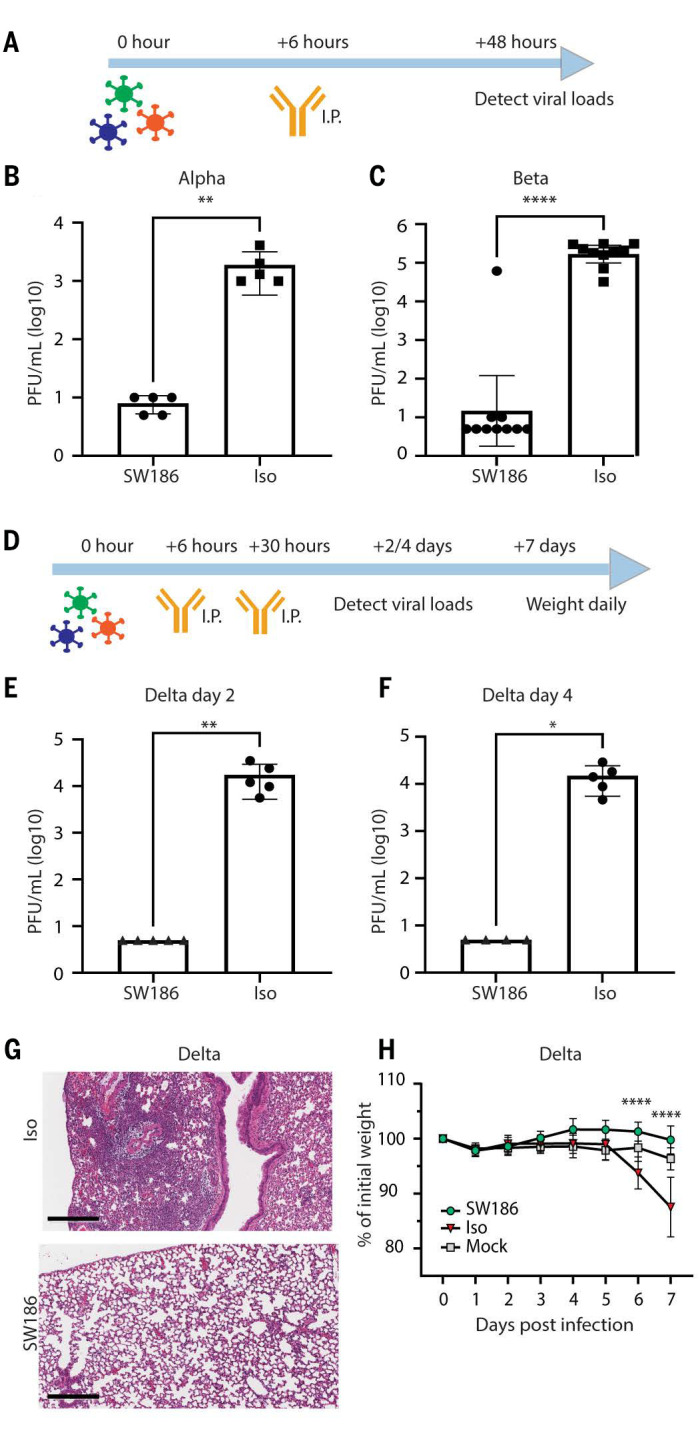
SW186 protects mice from SARS-CoV-2 infection. (A) The schematics of antibody treatment in mice infected with the Alpha and Beta variants of SARS-CoV-2. Mice were intranasally infected with the indicated SARS-CoV-2 variants of concern. After 6 hours, the SW186 antibody or its isotype control antibody (Iso) was intraperitoneally (I.P) injected into mice. Lungs were harvested 2 days after infection to measure the viral loads. (B) Viral loads measured by plaque assay on day 2 post SARS-CoV-2 Alpha infection (n = 5). Antibody dose: 2.8 mg/kg. (C) Viral loads measured by plaque assay on day 2 after SARS-CoV-2 Beta infection (n = 10). Antibody dose: 5 mg/kg. (D) The schematics of antibody treatment in mice infected with SARS-CoV-2 Delta virus. Human ACE2 transgenic mice were intraperitoneally injected with SW186 or its isotype control (3 mg/kg) at 6 hours and 30 hours after viral infection. (E-F) Viral loads were measured by plaque assay on day 2 (E) and day 4 (F) after SARS-CoV-2 Delta infection (n = 5). Mann Whitney test was used in A-F. (G) Histopathology analysis of lungs from SARS-CoV-2 Delta infected mice. Lungs harvested at 4 days post-infection were stained with hematoxylin and eosin (H&E). Scale bars, 300 μm. Representative images from five mice per group. (H) Mouse body weights were measured daily after SARS-CoV-2 Delta infection and antibody treatment. N=5 for each antibody-treated group; Mock: no virus infection, n = 10. Statistical significance was tested by two-way ANOVA. p-values: ≤ 0.05 (*), ≤ 0.01 (**), ≤ 0.001 (***), ≤ 0.0001 (****).

To test if SW186 has a therapeutic effect in vivo, we infected BALB/c mice intranasally (IN) with 10^4^ PFU of Alpha or Beta SARS-CoV-2 variants. Six hours after infection, SW186 or an isotype control antibody was intraperitoneally injected into the infected mice. Mice were sacrificed on day 2, and the lungs were harvested to measure the viral loads (Fig. 5A). Compared to the control antibody, the SW186 treatment reduced the lung viral loads of Alpha and Beta by ~100-fold and ~10,000-fold, respectively (Fig. 5B-C). To further determine the treatment effect of SW186 on the Delta variant, we infected human ACE2-expressing K18 mice with 10^3^ PFU of Delta variant. We switched to the K18 mice because Delta variant did not have the spike N501Y substitution that was required for robust replication in the BALB/c mice. The mice were treated with SW186 or the isotype control antibody at 6 and 30 hours after viral infection. The body weights of a cohort of the mice were measured daily for seven days. Another cohort of mice were sacrificed at 2 days and 4 days after infection to harvest the lungs for viral load measurements and histology (Fig. 5D). The mice treated with the control antibody had lung viral loads of ~10^4^ pfu/mL on day 2 and day 4 after infection, whereas the SW186 antibody treatment decreased the viral loads by ~1,000-fold on both dates (Fig. 5E and 5F). Histopathology analysis of the lung showed that the SW186 treatment protected the lungs from the viral damage and inflammatory cell infiltration (Fig. 5G). The SW186 antibody treatment also effectively protected the mice from bodyweight loss caused by the Delta virus infection (Fig. 5H). Together, these results showed that SW186 had a therapeutic effect against Alpha, Beta, and Delta variants in mice.

### Generation and characterization of humanized SW186

**Fig. 6.**
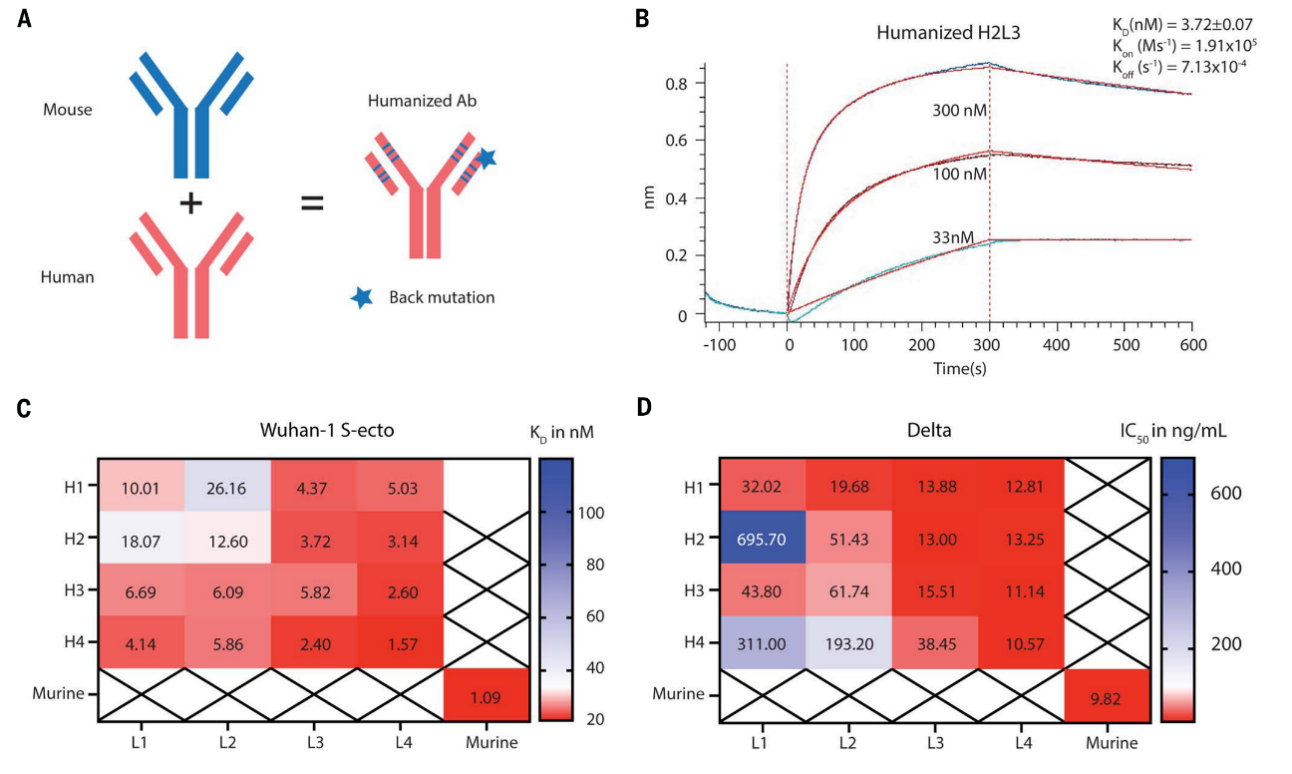
Humanization of SW186. (A) Illustration of mouse antibody humanization strategy. (B) The kinetics of humanized SW186 (H2L3) binding to the spike protein using BLI. The K_D_ value is mean ± SD, n=3. (C) This heatmap summarizes the mean K_D_ values of humanized SW186 antibodies binding to the spike protein of SARS-CoV-2 (Wuhan-hu-1 strain) as measured by BLI. The binding kinetic parameters are summarized in table S4. (D) Neutralization activities (IC_50_ at ng/mL) of humanized antibodies against SARS-CoV-2 Delta pseudovirus. Antibodies were serially diluted 1:3 from 30 μg/mL for 7 dilutions, with each dilution in duplicate.

In an effort toward testing SW186 in humans, we generated a panel of humanized SW186 by grafting the CDRs of SW186 into a human antibody vector with back mutations to restore the binding property (Fig. 6A). Four heavy chains and four light chains were designed, resulting in 16 antibodies with the combination of heavy and light chains (Table S3). We generated these antibodies and tested their binding to the spike protein. The results showed that all humanized antibodies retained their binding to the S-ecto protein and several antibodies such as H4L4 and H2L3 had K_d_ values similar to that of murine SW186 (Fig. 2A, Fig. 6B-C, Table S4). The binding of the humanized antibodies to the S-ecto protein was independently measured by ELISA (Fig. S11). We then tested the neutralizing activity of the humanized antibodies against the pseudovirus displaying the spike protein of the Alpha, Beta (Fig. S12) and Delta variants (Fig. 6D, Table S4). Consistent with the binding kinetics, most humanized antibodies neutralized these variants with IC_50_s similar to that of murine SW186. These humanized antibodies may be further engineered to improve the efficacy and breadth of inhibiting a variety of SARS viruses.

## DISCUSSION

In this article, we reported the isolation of a panel of SARS-CoV-2 antibodies from mice immunized with the viral spike protein. Among these, the SW186 monoclonal antibody showed the best neutralizing activity against all variants we have tested, including alpha, beta, delta, and the omicron subvariants. Remarkably, SW186 also effectively neutralized SARS-CoV-1. We determined the cryo-EM structure of SW186 Fab bound to the spike protein of SARS-CoV-2. The structure showed that SW186 bound to an outer surface of RBD, which was distinct from the RBM that bound to ACE2. The binding epitope of SW186 was highly conserved among SARS-CoV-1 and SARS-CoV-2 variants. Administration of the SW186 antibody into mice after they were infected with SARS-CoV-2 variants reduced the viral loads in the lung, demonstrating a therapeutic effect.

A significant feature of SW186 is that it bound to a highly conserved epitope outside the RBM, which is different from most current therapeutic antibodies (*10*, *19*–*23*). The RBM region is highly mutated in the Omicron variants, resulting in their immune escape of RBM-targeting antibodies. The conserved epitope of SW186 enabled this antibody to retain neutralizing activity against many SARS-CoV-2 variants we have tested so far. The epitope of SW186 includes several conserved amino acids, including N343 which is glycosylated and invariant among all SARS-CoV-2 viruses sequenced so far as well as the SARS-CoV-1 virus. This high degree of conservation of N343 implies an important function. Indeed, N343 glycosylation facilitates the opening of the RBD from a ‘down’ to ‘up’ state, which is required for ACE2 binding (*17*). Although SW186 did not bind to the RBM to directly interfere with ACE2 binding, it still partially inhibited the binding of the spike protein to ACE2. However, this partial inhibition appeared insufficient to explain the effective neutralizing activities of SW186 against multiple SARS-CoV-2 variants. An interesting possibility, which requires further investigation, is that the binding of SW186 inhibits the downstream events after the receptor binding, such as the virus entry into host cells. This mechanism may also explain the neutralizing effect of the S309 antibody, which also binds to a partially overlapping epitope that includes N343. The epitope on the RBD recognized by SW186 belongs to Class 3 category which binds outside of ACE2 binding interface (*24*), suggesting that additional antibodies may be developed that target this region.

It is remarkable that SW186 and S309 were generated against the spike proteins of SARS-CoV-2 and SARS-CoV-1, respectively, yet both antibodies have broad neutralizing activity against both classes of the virus by targeting similar, albeit non-identical, epitopes, suggesting that these epitopes are an Achilles heel for the SARS virus. Nevertheless, the decreased neutralization of the newer Omicron subvariants by both SW186 and S309 indicates that the virus can still generate new variants that evade antibody neutralization. Further structure-based engineering of SW186 may lead to development of more broadly effective antibodies against the circulating and future SARS viruses.

## MATERIAL AND METHODS

### Study Design

This study aimed to identify broadly neutralizing antibodies against SARS-CoV-2, especially currently circulating virus variants of concern. We used the LIBRA-seq (linking B cell receptor to antigen specificity through sequencing) technology to identify SARS-CoV-2 spike-specific B cells in immunized mice. We screened for broadly neutralizing antibodies based on a neutralization assay with multiple circulating SARS-CoV-2 variants. A broadly neutralizing antibody, SW186, was further tested for its ability to protect mice from multiple SARS-CoV-2 variants infection. The mode of binding of this antibody to the spike protein was determined by Cryo-EM and confirmed by structure-guided site-directed mutagenesis. The number of replicates and mice per experimental group was indicated in figure legends. The lung histopathology was assessed by a pathologist from the Pathology Core at UT Southwestern.

### Cloning, expression, and purification of Spike and hACE2 proteins

The pαH-Spike (1-1208)-T4Tri-HRV3Csite-TwinStrep-8XHis was a gift from Dr. Jason S. McLellan (*25*). cDNA encoding the spike ectodomain (S-ecto) was constructed based on the Wuhan-hu-1 sequence with a 2P mutation at residues 986 and 987, followed by an 8xHis Tag, a Strep-Tactin Tag and an AviTag (*25*). All mutants and Spike-RBD (319−527) were generated with PCR-based strategy. The spike and the Spike-RBD proteins were expressed in ExpiCHO-S cells with standard protocol (ThermoFisher). Briefly, cells were cultured to the density of 6.0x10^6^ at 37°C and transfected with 1 μg/mL plasmid DNA using ExpiFectamine CHO transfection Kit (ThermoFisher). 24 hours after transfection, the culture was supplemented with 0.12% (v/v) ExpiCHO enhancer and 5% (v/v) feed. The supernatant was harvested at Day 8 post-transfection and filtered with 0.22 μm membrane for further purification. HisTrap Excel column (Cytiva) was pre-equilibrated with wash buffer containing 25 mM Tris pH 8.0, 500 mM NaCl and 20 mM imidazole, and the filtered supernatant was then loaded onto the column. The resin was rinsed with 10 column volume (CV) of wash buffer and eluted with 5 CV of wash buffer plus 300 mM Imidazole. The eluent was collected, concentrated and applied to Superose 6 Increase 10/300 or Superdex 200 10/300 Increase (Cytiva) in a buffer containing 25 mM Tris pH 8.0 and 150 mM NaCl (SD buffer). The peak fractions were collected and flash-frozen in liquid nitrogen for further use.

For human ACE2 protein, the gene was synthesized and subcloned into a modified pCAG vector, with a His_6_ tag at C terminus (pCAG-His). The hACE2-His_6_ protein was expressed in Expi293 cells with the same transfection protocol using PEI 25K. Cells were harvested 60 hours after transfection using SD buffer supplemented with 1 mM PMSF and 1x protease inhibitor cocktail (EDTA-free, Roche) and disrupted by sonication, followed by 50,000 g centrifugation for 1 hour at 4°C. The supernatant was loaded onto Ni-NTA resin (Qiagen). The resin was rinsed with wash buffer for three times, and protein was eluted with wash buffer plus 300 mM Imidazole. After concentration, the protein was applied to Superdex 200 10/300 Increase in SD buffer. Peak fractions were collected for BLI-based competition assay.

### Antigen oligonucleotide barcode labeling and biotinylation

Oligo DNA barcodes were designed based on the LIBRA-seq protocol (*9*). In brief, a 15 nt DNA barcode was inserted into the following sequence: 5′-CCTTGGCACCCGAGAATTCCA-Barcode-CCCATATAAGA*A*A-3′. The barcodes used in this study were: TCCTTTCCTGATAGG(Spike), GCTCCTTTACACGTA(RBD). All the oligo barcodes were synthesized with 5′ amino modifier C6 and purified by HPLC (Integrated DNA Technologies; IDT). After purification, AviTag labeled proteins were biotinylated using the BirA biotin-protein ligase reaction kit (Avidity LLC, cat no. BirA500). The barcode oligos were linked to biotinylated S-ecto or RBD using the Solulink Protein-Oligonucleotide Conjugation Kit (TriLink cat no. S-9011) according to manufacturer’s instructions.

### Mouse immunization

Mouse immunization experiments were performed under specific pathogen-free conditions in the Animal Research Center at the University of Texas Southwestern Medical Center according to protocols approved by the Institutional Animal Care and Use Committee. 8-week-old female wild-type C57BL/6J mice were purchased from the Jackson Laboratory and immunized with 5 μg RBD or 10 μg S-ecto (Wuhan-hu-1 strain) as the antigen and 10 μg 2’3′cGAMP as the adjuvant via intramuscular injection (IM). Mice were boosted with the same immunization protocol twice at 4 weeks (IM) and 6 weeks (intravenously) after the initial immunization. And three days after the second boosting, mice were sacrificed and the spleen cells were collected for memory B cell isolation.

### Memory B cell enrichment and antigen specific B cell isolation

After red blood cell lysis and blocking with anti-CD16/CD32(BioLegend, Cat#101302), memory B cells were enriched with Memory B Cell Isolation Kit (Miltenyi Biotec, Cat#130-095-838) following standard protocol. In brief, non-B cells were labeled with a cocktail of biotin-conjugated antibodies and depleted with Anti-Biotin MicroBeads. Then, IgG1/IgG2 expressing cells were positively selected with IgG1-APC/IgG2-APC and Anti-APC Microbeads. These cells were stained with a biotinylated and DNA barcode labeled antigen (5μg/mL) on ice for 30 min. After washing, cells were further stained with PE-streptavidin for 15 min. After further washing, cells were resuspended in MACS buffer (1% FBS, 1 mM EDTA in PBS) with Propidium Iodide. Cells were sorted on Aria 1 flow sorter (BD Biosciences) to enrich antigen-specific memory B cells. Cells from naive mice served as a negative control. Cells sorted with different antigens were combined and processed for single cell V(D)J library construction immediately.

### Single cell sequencing library preparation and next generation sequencing

Single cell V(D)J library was constructed following 10x Genomics User Guide (CG000186 Rev D). The antigen DNA barcode library was constructed following CITE-seq protocol (https://cite-seq.com/protocols/). ADT PCR additive primer (5′- CCTTGGCACCCGAGAATT*C*C-3′) was added into cDNA mix to amplify DNA barcode oligos. Both V(D)J and antigen DNA barcode library were sequenced by Novogene and resulted in 29,458 paired reads per cell in V(D)J library.

### Antigen specific B cell receptor transcripts identification

V(D)J library reads were processed using cellranger 4.0.0 with GRCm38 Reference - 4.0.0. The antigen DNA barcodes were recovered using CITE-seq-Count (*26*). Only B cells with both productive BCR heavy and light chains, and DNA barcode information were selected for further data analysis. B cells with both Lambda and Kappa light chains were removed since they might not represent single cells. Qualified B cells were grouped into clones using Change-O (*27*) 10x pipeline (https://changeo.readthedocs.io/en/stable/examples/10x.html) with the Hamming distance model. The cutoff of nearest neighbor distance (0.08) was determined with shazam (*27*) R package through calculating the distribution of pairwise Hamming distances between the sequences. B cell antigen specificity was determined based on antigen DNA barcode’s unique molecular identifier, or UMI. B cells with ≥ 100 antigen barcode UMI were defined as B cells specific to this antigen (background UMI median = 2). Clones containing more than two B cells specific to the same antigen were defined as clonally expanded B cell clones.

### Expression and purification of recombinant monoclonal antibodies

cDNAs encoding the BCR heavy and light variable regions were synthesized by GeneScript and subcloned into pFUSE-CHIg-HG1 and pFUSE2ss-CHIg-hK vectors (InvivoGen), respectively. Full-length antibodies were expressed using ExpiCHO cells according to standard protocol. Cells were transfected with 0.6 μg/mL light chain plasmid and 0.4 μg/mL heavy chain plasmid. The medium was collected at Day 8 post-transfection. Antibodies from the medium were purified using Pierce Protein A/G Agarose (ThermoFisher, Cat# 20424) with the standard protocol. Filtered medium (0.22 μm) was loaded onto Protein A/G Agarose pre-equilibrated with binding buffer (25 mM Tris pH 8.0, 150 mM NaCl, 1 mM EDTA). After washing with the same buffer for three times, the protein was eluted with 5 CV of 0.1 M Glycine pH 2.7 and the pH of eluent was immediately adjusted with 0.5 CV 1 M Tris pH 8.0. Protein was concentrated and further applied to Superdex 200 10/300 Increase (Cytiva) in Dulbecco’s PBS (Sigma) buffer. For SW186 Fab protein, the cDNA of heavy chain and light chain was subcloned into modified pCAG-His vector with an IL2 signal peptide at N terminus. A stop codon was introduced to the C terminus of light chain to make it a tag-free protein. The protein was then expressed the same way as full-length antibodies, and purified with HisTrap Excel column (Cytiva) using the AKTA system.

### Sample preparation of Spike-SW186 Fab complex for Cryo-EM and data collection

Purified spike ectodomain and SW186 Fab protein were mixed with the molar ratio of 1:4, and the mixture was incubated for 2 hours at 4°C. After incubation, the sample was applied to Superose 6 10/300 Increase in SD buffer to remove excess Fab. Peak Fractions were collected and concentrated to 0.7 mg/mL, then applied to glow-discharged Quantifoil R1.2/1.3 300-mesh gold holey-carbon grids (Quantifoil, Micro Tools GmbH, Germany). Grids were blotted for 4 s under 100% humidity at 4°C before being plunged into the liquid ethane using a Mark IV Vitrobot (FEI). Micrographs were acquired on a Titan Krios microscope (FEI) operated at 300 kV with a K3 direct electron detector (Gatan), using a slit width of 20 eV on a GIF-Quantum energy filter. SerialEM was used for the data collection. A calibrated magnification of 46,296 was used for imaging of the samples, yielding a pixel size of 1.08 Å on images. The defocus range was set from -1.6 μm to -2.6 μm. Each micrograph was dose-fractionated to 30 frames with a total dose of about 60 e-/Å^2^.

### Image processing

The cryo-EM refinement statistics are summarized in Table S2. 3,806 movie frames were motion-corrected and binned two-fold, resulting in a pixel size of 1.08 Å, and dose-weighted using MotionCor2 (*28*). The CTF parameters were estimated using Gctf (*29*). RELION3 (*30*) was used for the following processing. Particles were first picked using the Laplacian-of-Gaussian blob method, and then subjected to 2D classification. Class averages representing projections of spike/Fab protein in different orientations were used as templates for reference-based particle picking. Extracted particles were binned three times and subjected to 2D classification. Particles from the classes with fine structural feature were selected for 3D classification using an initial model generated from a subset of the particles in RELION3. Particles from one of the resulting 3D classes showing good secondary structural features were selected and re-extracted into the original pixel size of 1.08 Å. Subsequently, we performed finer 3D classification imposed by using local search in combination with small angular sampling, resulting in new classes with 1 or 2 Fab bound at RBD domains. The cryo-EM map after 3D refinement of spike with one Fab bound was resolved at 3.3 Å resolution, but the RBD-SD1 and the bound Fab appeared blurred, suggesting flexibility between RBD-SD1 and the rest of spike. To improve the resolution, we performed focused refinement with density subtraction on the RBD-SD1 and Fab. The modified particle set was subjected to another round of 3D refinement with a soft mask, leading to a markedly improved density for the Fab bound RBD-SD1 at 3.4 Å resolution.

### Model building and refinement

Initial model of SW186 Fab generated by AlphaFold2 engine (*31*) and RBD-SD1 (PDB code: 6XR8) was docked into the locally refined map using PHENIX (*32*). Coot (*33*) was used for fitting the atomic model into the map. The final model of SW186 Fab bound to RBD-SD1 was refined and validated by PHENIX. All structural models are presented using PyMOL (*34*).

### ELISA

To measure the binding of antibodies with target antigens, 50 ng of S-ecto or RBD was coated on each well of the 96-well ELISA plate at 4°C overnight. After washing with PBST (PBS containing 0.05% tween-20), plates were incubated with a blocking buffer (1% BSA in PBS) at room temperature for 2 hours. Antibodies at different dilutions were added to the plates and incubated at room temperature for 1.5 hours. After washing with PBST, plates were incubated with anti-human-IgG-HRP (Jackson ImmunoResearch, Cat# 109-035-088) (1:5,000 dilution) for 45 min. Plates were washed with PBST 5 times between two steps. Finally, TMB substrate solution (ThermoFisher, Cat# N301) was incubated with the plate for 15 min before addition of a stop solution (1.8 N H_2_SO_4_). Absorbance at 450nm was measured using CLARIOstar Plus Microplate Reader (BMG labtech).

### Pseudovirus generation

SARS-CoV-2 pseudoviruses were generated according to a published method (*35*). The coding sequence for Wuhan-hu-1 spike and a C-terminal His_6_ Tag was inserted into the pcDNA3.1(+) (ThermoFisher) vector for pseudovirus packaging. HIV-1 pseudovirus displaying a SARS-CoV-2 spike protein was produced in HEK293T cells (ATCC) by co-transfecting pcDNA3.1-Spike with HIV-1 NL4-3 ∆Env ∆Vpr Luciferase Reporter Vector (*10*) using Lipofectamine 2000 (ThermoFisher). 12 hours after transfection, medium was replaced with fresh DMEM medium (1% (v/v) Antibiotic-Antimycotic, 10% (v/v) FBS). 60 hours after transfection, the supernatant was centrifuged at 200 g for 10 min, and passed through a 0.45 μm filter. The filtered solution containing the virus was aliquoted and frozen at −80°C. Mutant pseudoviruses were produced by site-directed mutagenesis on the original pcDNA3.1-Spike vector. The mutations are shown in Fig. S3. SARS-CoV-1 and WIV1 pseudoviruses were generated using the same method as SARS-CoV-2 pseudoviruses except that pcDNA3.1-SARS-Spike (Addgene 145031), pcDNA3.3-CoV-1-D28 (Addgene 170447), or pTwist-WIV1-CoV (Addgene 164438) was used instead of SARS-CoV-2 Spike plasmid.

### Pseudovirus neutralization assay

The pseudovirus neutralization assay was performed using Huh-7 cells stably expressing hACE2. The cells (100 μL, 3,000 in DMEM) were seeded on a 96-well plate overnight. Various concentrations of mAbs (4-fold serial dilutions starting at 30 μg/mL, 50 μL aliquots in triplicates) were mixed with the same volume of SARS-CoV-2 pseudovirus in a 96 well-plate. The mixture was incubated for 1 hour at 37°C with 5% CO_2_. No-virus control wells were supplied with 100 μL DMEM medium (1% (v/v) Antibiotic-Antimycotic, 25 nM HEPES, 10% (v/v) FBS). Virus-only control wells contained 50 μL medium and 50 μL pseudovirus. After 1 hour, medium was removed from Huh-7 cells, and then 100 μL pseudovirus and antibody mixture was incubated with the cells for 1 hour at 37°C with 5% CO_2_. Another 100 μL DMEM medium was added into each well and incubated with the cells for 48 hours at 37°C with 5% CO_2_. After the incubation, supernatants were removed, and 100 μL Nano-Glo® Luciferase Assay Reagent (Promega) (1:1 diluted in PBS) was added to each well and incubated for 5 min. Luminescence was measured using CLARIOstar Plus Microplate Reader (BMG labtech). The relative luciferase unit (RLU) was calculated by normalizing luminescence signal to the virus-only control group. IC50 was determined by a four-parameter nonlinear regression using GraphPad Prism 9.0 (GraphPad Software Inc.).

### Recombinant SARS-CoV-2 viruses

The recombinant SARS-CoV-2 spike variants were constructed in the genetic background of an infectious cDNA clone derived from clinical strain WA1 (Wuhan-hu-1/USA_WA1/2020) with or without a mNeonGreen reporter (mNG) (*11*). The following viruses were used in this study: the alpha, beta, delta-spike SARS-CoV-2 were used for mouse infection experiments (*36*, *37*); SARS-CoV-2 mNG spike variants including alpha, beta, delta (*38*) and omicrons BA.1, BA.2.12.1 and BA.4/5 (*39*–*41*) were used for virus neutralization assays. All experiments involving infectious SARS-CoV-2 were performed in a BSL3 facility at the University of Texas Medical Branch (UTMB) according to approved protocols.

### Recombinant virus neutralization assay

Neutralization titers of monoclonal antibodies were determined by a fluorescent focus reduction neutralization test (FFRNT) using the mNG reporter SARS-CoV-2 described previously (*39*) with some modifications. Briefly, Vero E6 cells (2.5 × 10^4^) were seeded in each well of black μCLEAR flat-bottom 96-well plate (Greiner Bio-one). The cells were incubated overnight at 37°C with 5% CO2. On the following day, antibodies were 2 or 3-fold serially diluted in the culture medium and incubated with 100-200 fluorescent focus units (FFU) of mNG SARS-CoV-2 at 37°C for 1 hour, after which the antibody-virus mixtures were inoculated onto the pre-seeded Vero E6 cell monolayer in 96-well plates. After 1 hour infection, the inoculum was removed and 100 μL of overlay medium (DMEM supplemented with 0.8% methylcellulose, 2% FBS, and 1% penicillin and streptomycin) was added to each well. After incubating the plates at 37°C for 16 hours, raw images of mNG fluorescent foci were acquired using CytationTM 7 (BioTek) armed with 2.5× objective and processed using the default software setting. The foci in each well were counted and normalized to the non-antibody-treated controls to calculate the relative infectivity. The curves of the relative infectivity versus the serum dilutions were plotted using Prism 9 (GraphPad). A nonlinear regression method was used to determine the dilution fold that neutralized 50% of mNG SARS-CoV-2 (defined as FFRNT50). The SW186 antibody was tested in duplicates.

### Cell-based hACE2 competition assay by flow cytometry

The cell-based hACE2 competition assay was performed using ACE2-Expressing Huh-7 cells (Huh-ACE2). A pilot experiment showed that, at 5 μg/mL, S-ecto protein bound to ~80% Huh-ACE2 cells in the absence of a competing antibody. In the competition assay, Huh-ACE2 cells were incubated with anti-human CD16/32 for 20 min on ice to prevent non-specific binding of human IgG constant region. Then, biotinylated S-ecto (5 μg/mL) was pre-incubated with serially-diluted SW186 (0, 1, 5, 10, 15, 30, 50 μg/mL) or a control antibody as indicated for 1 hour at room temperature (RT). After washing with MACS buffer, the mixture was incubated with Huh-ACE2 cells for 30 min at RT. Cells were washed with MACS buffer twice, followed by staining with FITC- streptavidin at 2.5 μg/mL for 20 min on ice. After further washing, cells were analyzed by flow cytometry (Cytek Aurora, 5-laser). CC12.3 (*16*), an antibody known to compete with hACE2, was used as a positive control. 279_130, an antibody that binds to the spike protein outside the RBD (Fig. 1B), was used as a negative control. The relative competition percentage = $\left(1-\frac{S+\%\;in\;sample}{S+\%\;in\;negative\;control}\right)\times 100\%$ . Data were fitted with a four-parameter nonlinear regression model.

### Biotinylation of protein for BioLayer Interferometry (BLI)

S-ecto, hACE2-Fc, and Bovine serum albumin (BSA) proteins were biotinylated using EZ-link biotinylation Kit (NHS-sulfo-LC-Biotin, ThermoFisher) with standard protocol. Briefly, 1 mL protein (2 mg/mL) in PBS was incubated with 20-fold molar excess of freshly prepared reagent for 30 min at RT. The reaction was stopped by 0.1 M glycine and then applied to Zeba spin desalting column (7k MWCO, 2 mL, ThermoFisher) in PBS. The eluent was used for BLI assay.

### BLI analysis of SW186

The BLI-based kinetic assay of SW186 was performed according to a published protocol (*42*). Briefly, the kinetic buffer containing PBS plus 0.02% Tween-20 and 0.1% (w/v) BSA was used for calculating the baseline. To measure the kinetics of SW186 Fab binding to spike, streptavidin sensor (SA, Sartorious, Lot No. 2108010611) was pre-hydrated and incubated with 100 nM biotinylated S-ecto or biotinylated BSA as a negative control. SW186 Fab diluted with kinetic buffer was then applied to the sensor followed by measurement of the binding kinetics using Octet R8 (Sartorius). For ACE2 competition assay, 100 nM biotinylated hACE2-Fc was loaded onto pre-hydrated SA sensor for 4 min at 18°C. SW186 Fab or hACE2-His (as a positive control) was pre-incubated with 100 nM S-ecto at RT for 30 min. The mixture was applied to the SA sensor bound to hACE2-Fc and the binding kinetics was analyzed at 18°C, with 5 min association and 5 min dissociation. The spin speed for all experiments was 1,000 rpm.

### Antibody treatment of mice infected with SARS-CoV-2

The viral infection study in mice was carried out following the recommendations for care and use of animals by the Office of Laboratory Animal Welfare, National Institutes of Health. The Institutional Animal Care and Use Committee (IACUC) of the University of Texas Medical Branch (UTMB) approved the animal studies under protocol 2103023. Two studies were performed as follows.

*Infection with alpha and beta variants:* Ten-week-old female BALB/c mice were purchased from Charles River (Wilmington, MA, USA). The mice (n=5) were infected intranasally (IN) with 10^4^ PFU of alpha or beta-spike SARS-CoV-2 variants in 50 μL of PBS. Animals were injected intraperitoneally (i.p.) with SW186 or isotype control at 6 hours after viral infection. Two days after infection, lung samples of infected mice were harvested and homogenized in 1 mL PBS using the MagNA Lyser (Roche Diagnostics). The homogenates were clarified by centrifugation at 10,000 rpm for 3 min. The supernatants were collected for measuring infectious viruses by plaque assay on Vero E6 cells using a protocol described previously (*43*).

*Infection with the delta variant*: Eight-week-old female K18-hACE2 mice were ordered from The Jackson Laboratory. The mice were infected intranasally with 10^3^ PFU of SARS-CoV-2 Delta spike variant (*37*) in 50 μL of PBS. A moderate dose (10^3^ pfu) was chosen in infection of K18-ACE2 mice to avoid early death of the mice so that their body weights can be measured daily for 7 days. Animals were injected intraperitoneally (i.p.) with SW186 or isotype control antibody at 6 hours and 30 hours after viral infection. Two and four days after infection, lung samples of a cohort of infected mice were harvested and homogenized in 1 mL PBS. Viral titers were measured by plague assay as above.

### Histopathology analysis of the mouse lung

Four days after SARS-CoV-2 infection, lung samples of infected mice were harvested and fixed with 10% neutral buffered formalin. The fixed tissues were submitted to the Pathology Core at UT Southwestern for further dehydration, paraffin embedding, and Hematoxylin and Eosin (H&E) staining (*44*).

### Statistical analysis

Mann Whitney test or two-way analysis of variance (ANOVA) were used to assess the significance of difference as indicated in the figure legends. Two-sided testing was used with alpha of 0.05. IC_50_ values of ELISA and viral neutralization experiments were derived from antibody titration data by fitting with the four-parameter nonlinear regression in GraphPad Prism 9.0.
