# Supplementary material for: An Antibody That Neutralizes SARS-Cov-1 and SARS-Cov-2 by Binding To a Conserved Spike Epitope Outside the Receptor Binding Motif
Source: Sci Immunol. Author manuscript; Available in PMC 2022 Oct 30. (PMC9407945; doi:10.1126/sciimmunol.abp9962)
Supplement: Supplementary materials [file NIHMS1830525-supplement-Supplementary_materials.docx]

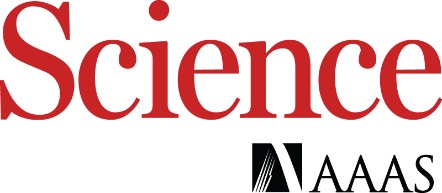


Supplementary Materials for

**An Antibody Neutralizes SARS-Cov-1 and SARS-Cov-2 by Binding To a Conserved Spike Epitope Outside the Receptor Binding Motif**

Yan Fang, Pengcheng Sun, Xuping Xie, Mingjian Du, Fenghe Du, Jianfeng Ye, Bo Li, Xiao-chen Bai, Birte K. Kalveram, Jessica A. Plante, Kenneth S. Plante, Pei-Yong Shi, Zhijian Chen

Correspondence to: Zhijian.Chen@UTSouthwestern.edu or peshi@utmb.edu

**This PDF file includes:**

Figure S1 to S12

Table S1 to S4


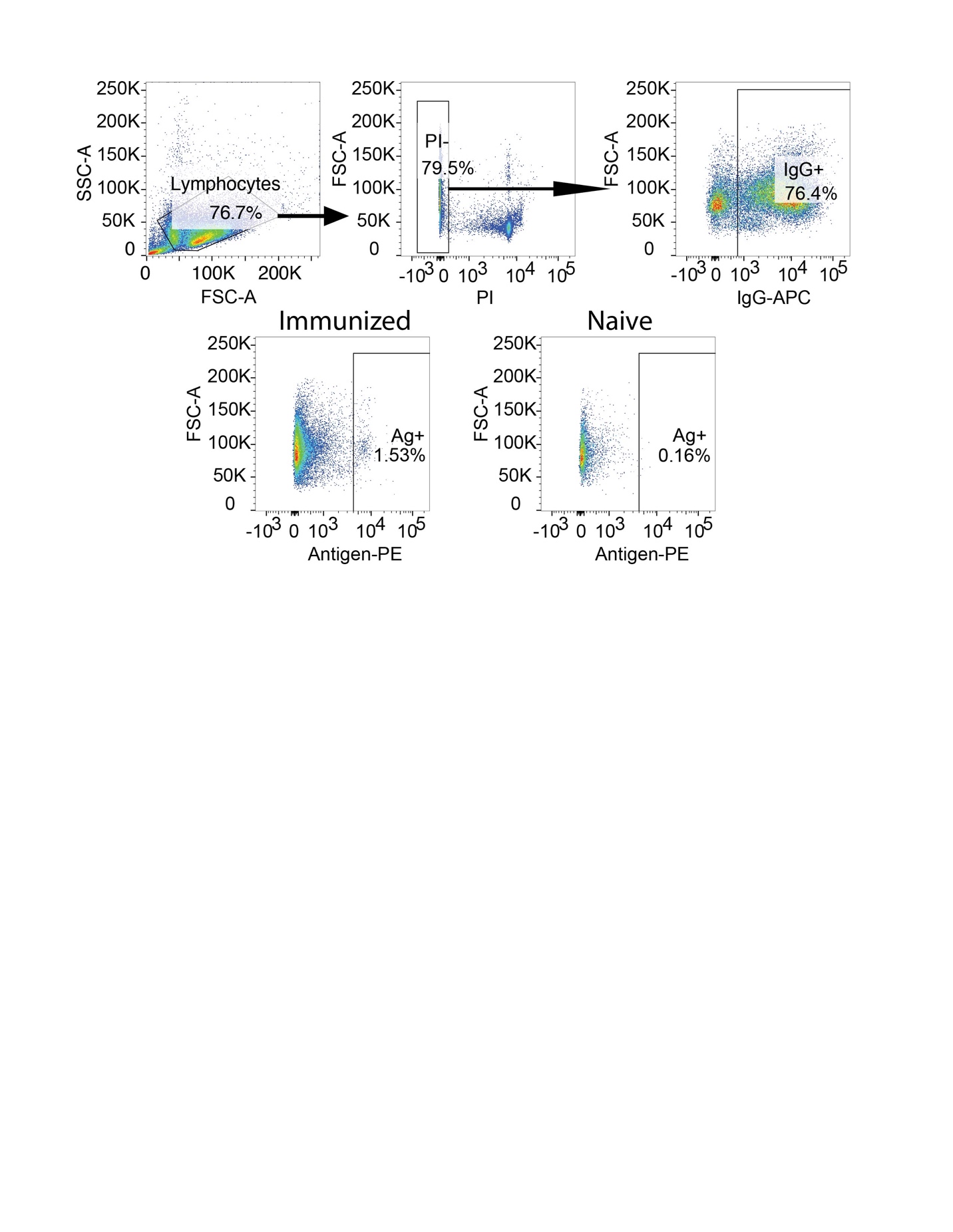


**Fig. S1. The gating strategy of flow cytometry**. In the PI- live cell gate, IgG-APC positive and PE-strep positive splenocytes (antigen positive) were sorted out. The splenocytes from naive (unimmunized) mice stained with the same regents show the background of this staining.


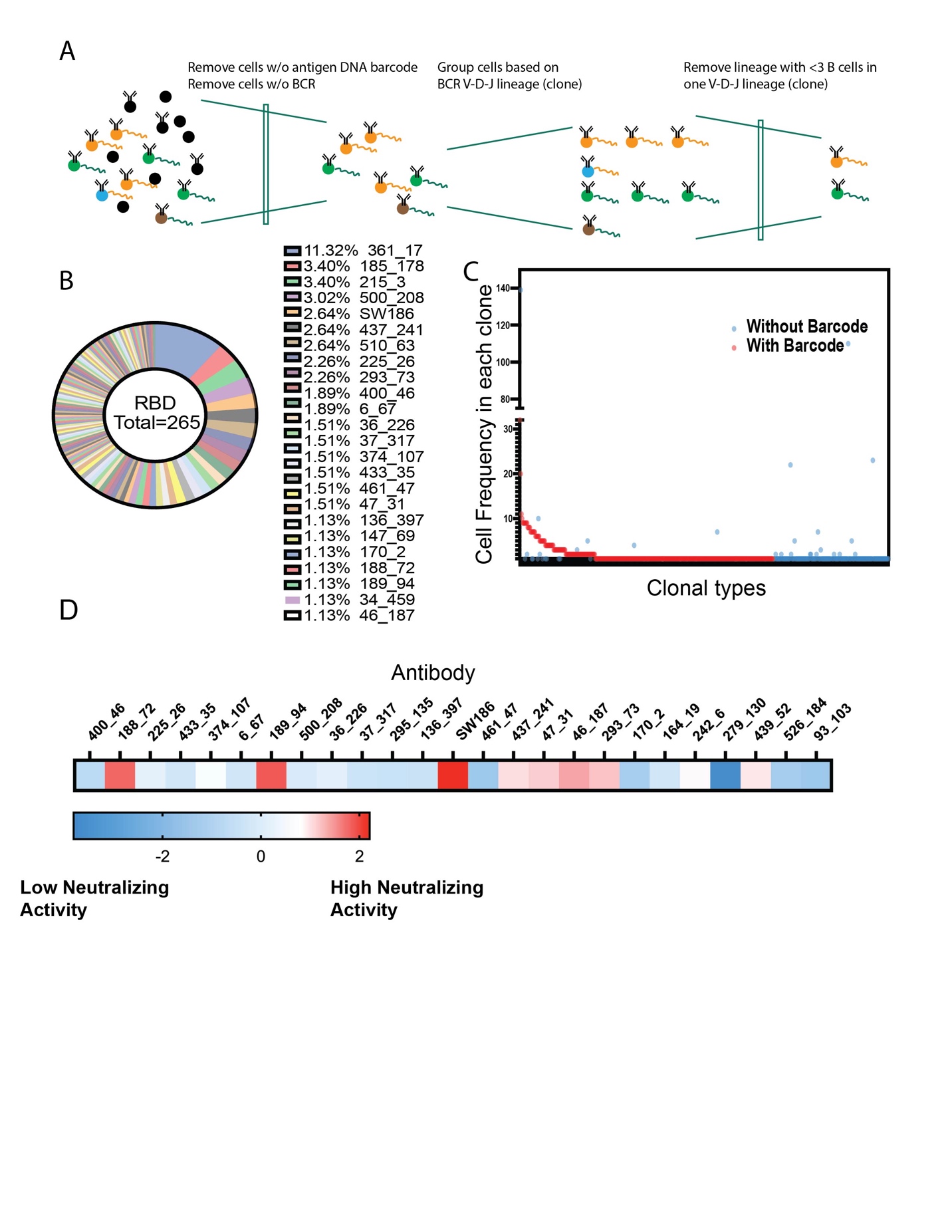


**Fig. S2. The process of antibody filtering.** (A) Illustration of antibody filtering during data analysis. The barcode negative or non-BCR cells were removed from the database. B cells were grouped into clones based on their germline sequences. Only B cells from expanded clones (B cells > 3 in each clone) are defined as antigen specific B cells and processed for further validation. (B) Clonal distribution of Spike-RBD specific BCRs. The percentage of expanded clones and their names are annotated on the right side of the figure. (C) The frequency of each B cell clone with (red) and without (blue) DNA barcodes. (D) The neutralizing activity (-log IC_50_ in ng/mL) of each antibody against the pseudovirus displaying the spike protein from the Wuhan-hu-1.


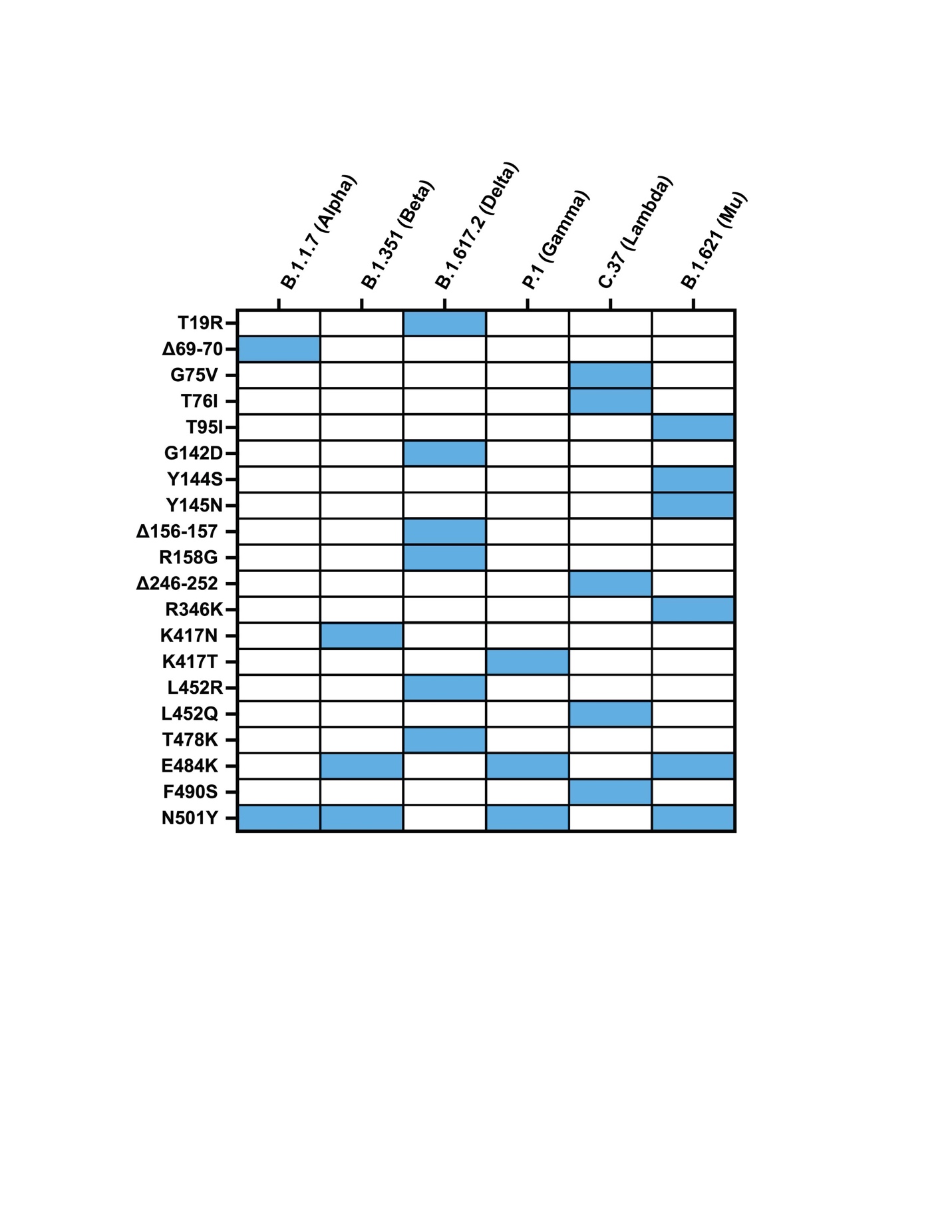


**Fig. S3. The mutation chart of spike protein on pseudoviruses.** To generate pseudoviruses variant to mimic SARS-CoV-2, we generated key mutations on spike proteins (labeled in blue) based on SARS-CoV-2 variant sequences.


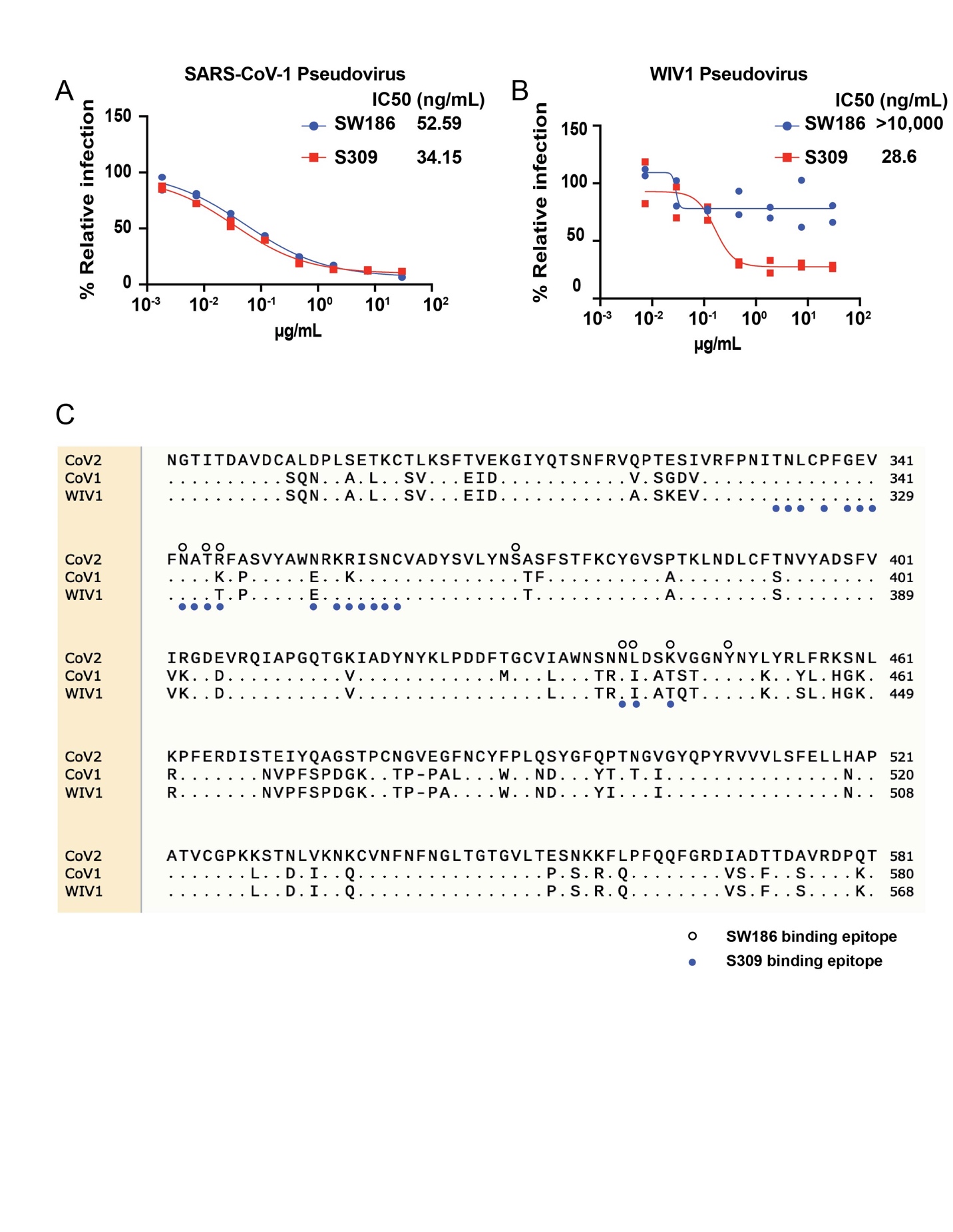


**Fig. S4. Neutralization of pseudovirus displaying SARS-CoV-1 or WIV1 spike protein**. Neutralization assays were performed using pseudotyped viruses displaying the SARA-CoV1 spike protein (A) or WIV1 spike protein (B). N=2. (C) The alignment of SARS-CoV-2, SARS-CoV-1, and WIV1 Spike proteins (focusing on RBD region). The binding epitopes of SW186 are indicated by black circles. The epitope of S309 are labeled based on literature (13).


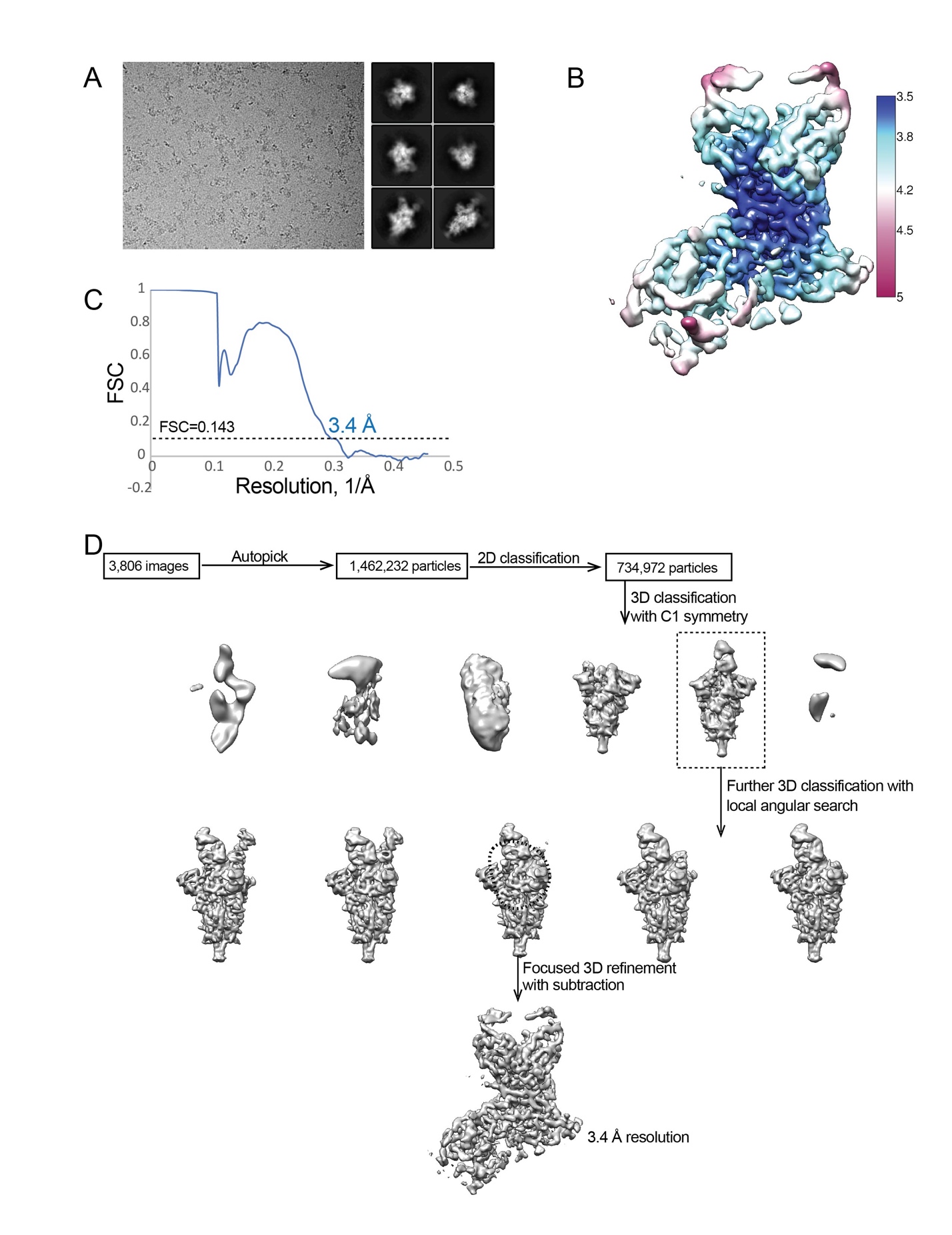


**Fig. S5. Image processing of SW186 Fab-RBD-SD1 complex**. (A) Cryo-EM micrograph (left) and 2D classification of SW186 Fab-spike. (B) Resolution map of locally refined Fab-RBD-SD1 map. (C) Gold-standard Fourier shell correlation curve for the local refinement. (D) Flowchart for image processing.


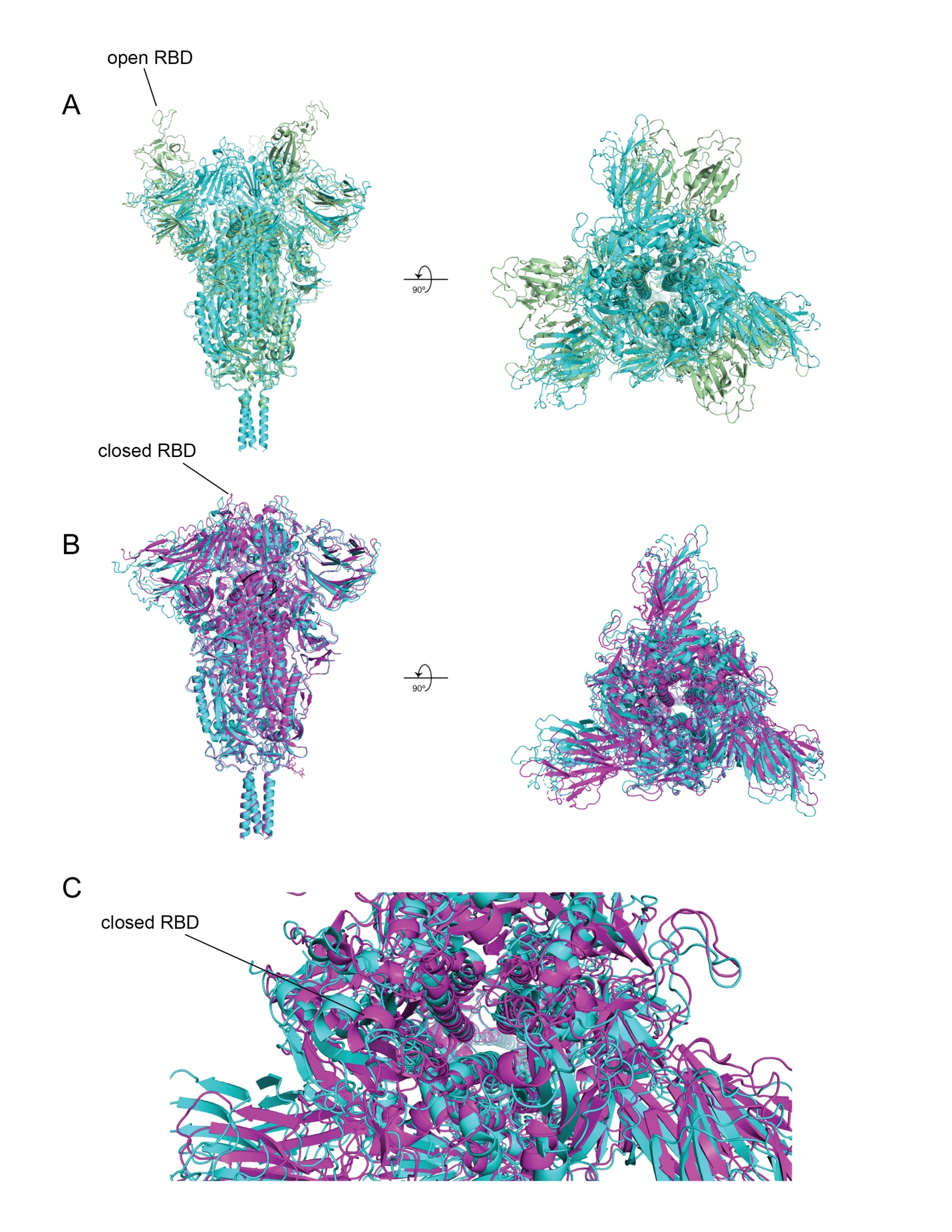


**Fig. S6. Superposition of SW186 Fab-bound full length spike in different states**. Both open (A; PDB code: 7CAK) and closed (B; PDB code: 6XR8) state models of spike are superimposed to SW186 Fab-bound Spike. Structure models are colored magenta, while the map is colored light grey. RBDs on the models are shown by lines. (C) Enlarged view of the close state model of spike shown in (B).


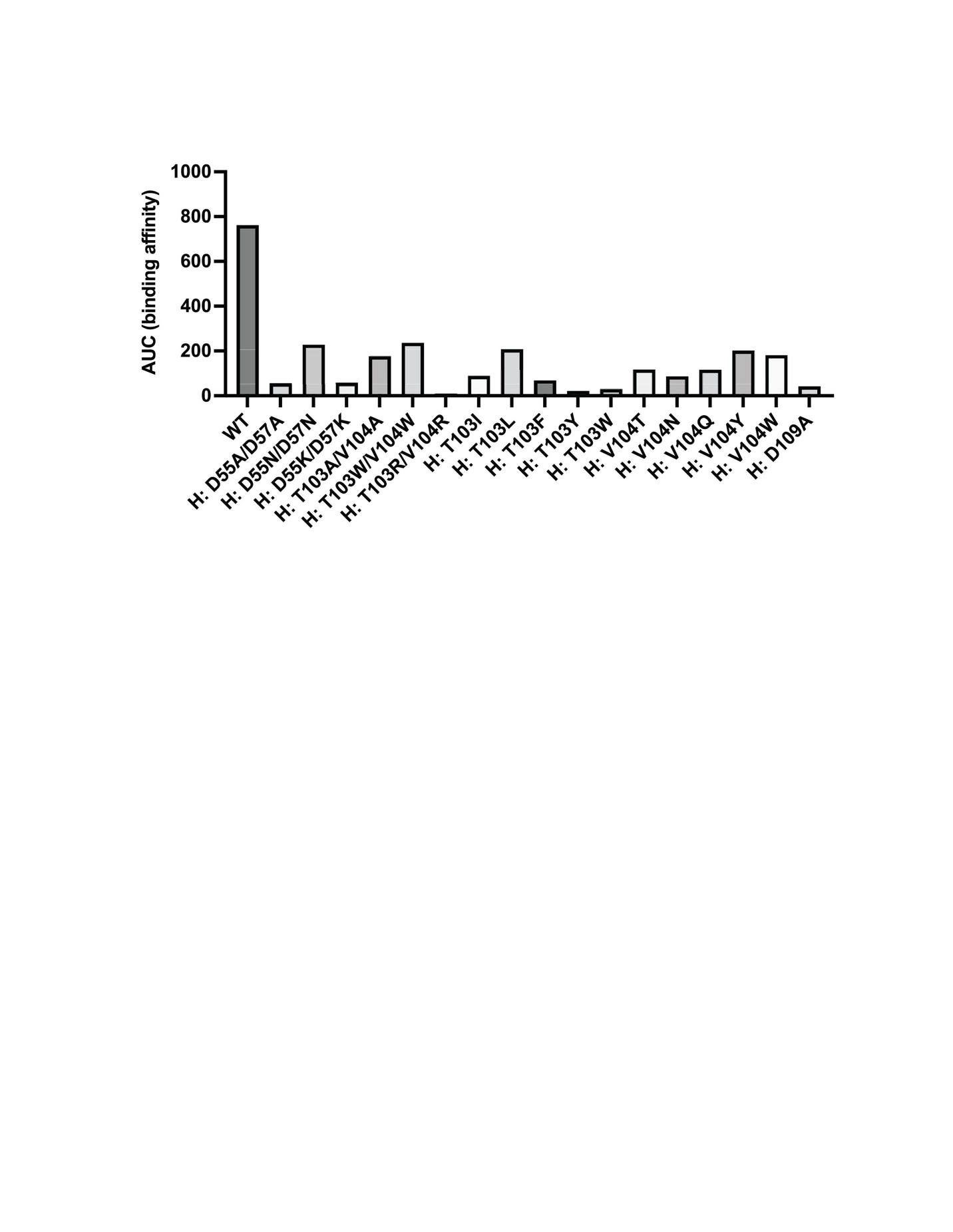


**Fig. S7. Structure-guided mutagenesis of SW186 and the effects on its binding to the spike protein.** The area under the curve (AUC) values were measured by ELISA assay. The unit of AUC is (μg/ml) * OD450.


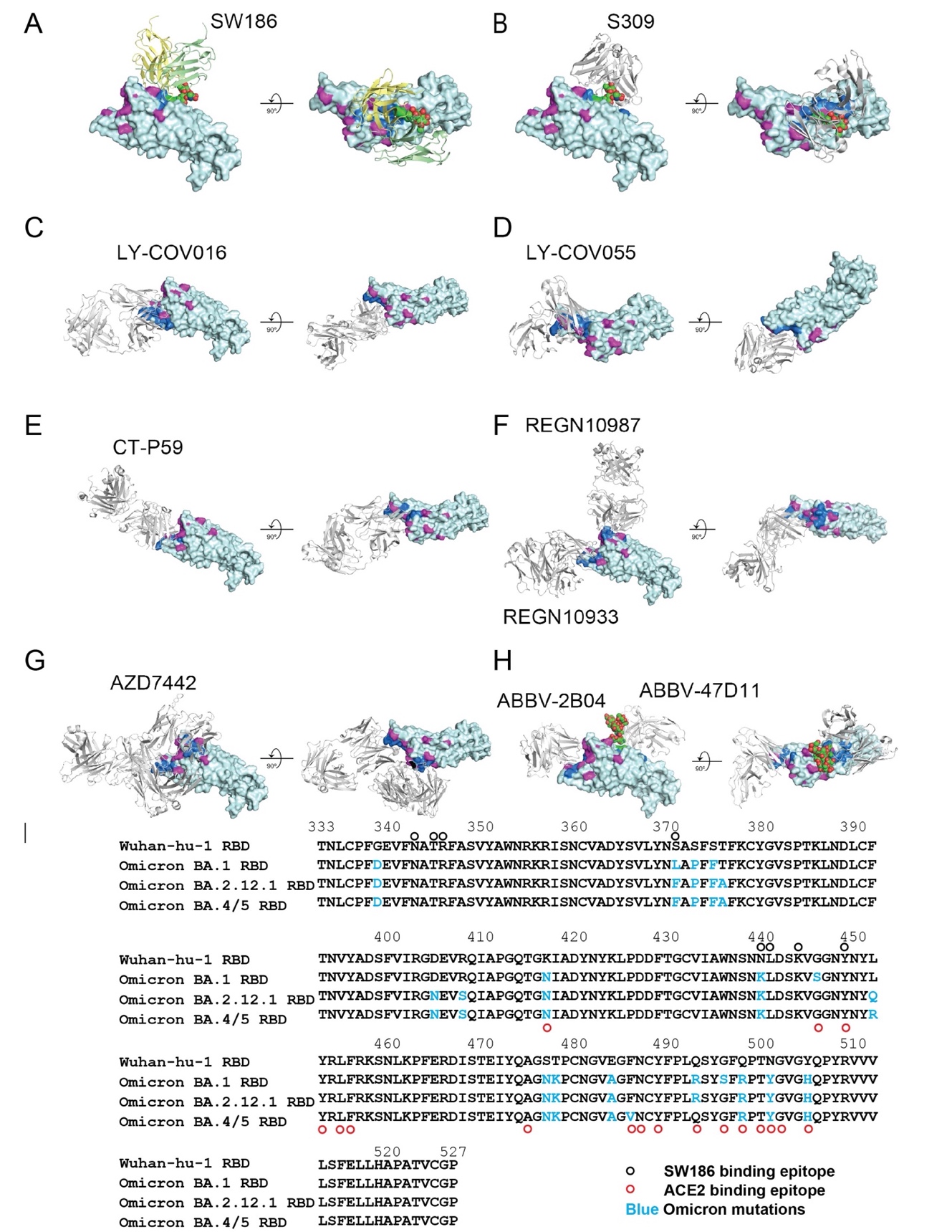


**Fig. S8. SW186 binds a conserved epitope on spike protein.** (A-H) Structural models of the Omicron RBD (pale cyan) bound to SW186 (A; V_H_ and V_L_ are colored pale yellow and pale green) and therapeutic antibodies (B-H; grey). Antibodies are shown as cartoon, and the RBD is presented as surface. The epitope of each antibody is colored marine, and the mutation sites of Omicron are colored magenta, including the overlap with the epitopes. N343-linked glycan is shown as green spheres in A and B. PDB codes of the structures in these panels are: S309: 6WPS, LY-COV016: 7C01, LY-COV555: 7L3N, CT-P59: 7CM4, REGN mAbs: 6XDG, AZD7442: 7L7E, ABBV mAbs: 7AKJ, 7K9H. (I) Sequence alignment of RBDs from the Wuhan-hu-1 and Omicron subvariants of SARS-CoV-2. The mutation sites on Omicron are shown in light blue. The binding epitopes of SW186 and hACE2 are indicated by black and red circles, respectively.


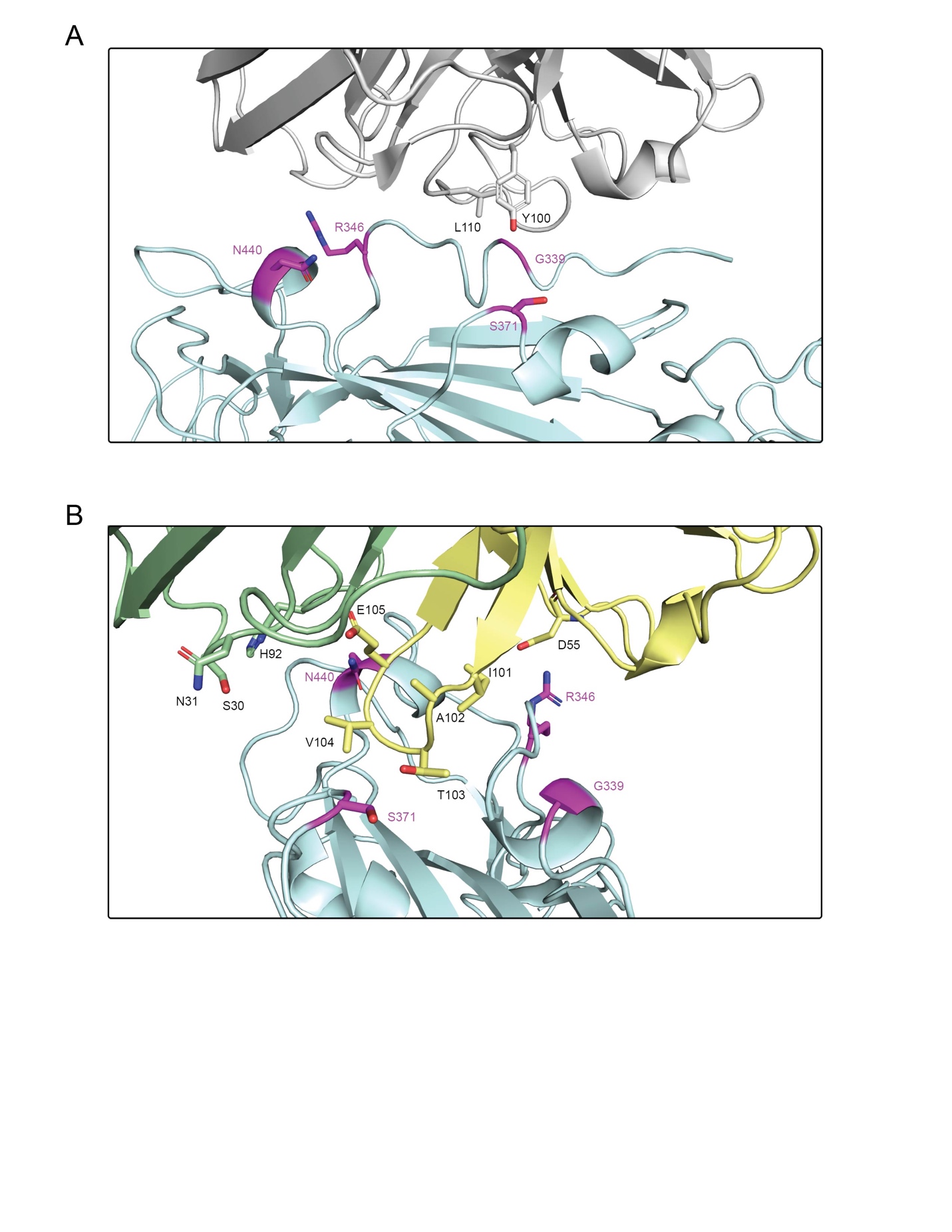


**Fig. S9. Comparison between S309 and SW186 with key mutations on omicron.** A zoom-in view of the antibodies S309 (A) and SW186 (B) binding to RBD. Mutations that largely decrease the neutralization activity of S309 are colored magenta. S309 and RBD are colored grey and pale cyan. V_H_ and V_L_ of SW186 are colored pale yellow and pale green. Surrounding residues are shown as sticks.

**
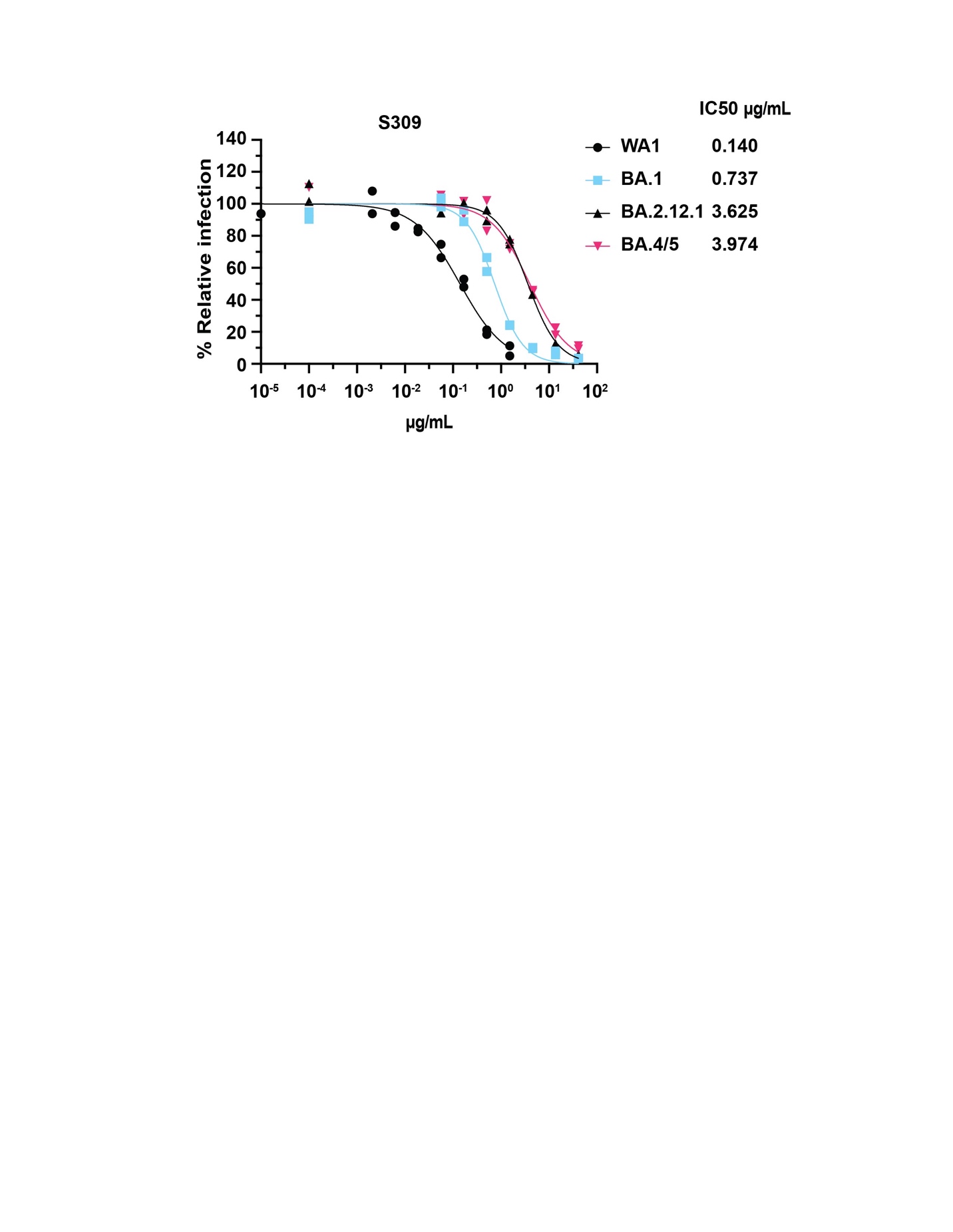
**

**Fig. S10. The neutralization activity of S309 against Omicron variants.** Neutralization assays were performed as in Figure 2 using recombinant SARS-CoV-2 omicron subvariants as indicated. Serial dilutions of the antibodies were done in duplicates. A four-parameter nonlinear regression was used to calculate the IC_50_.


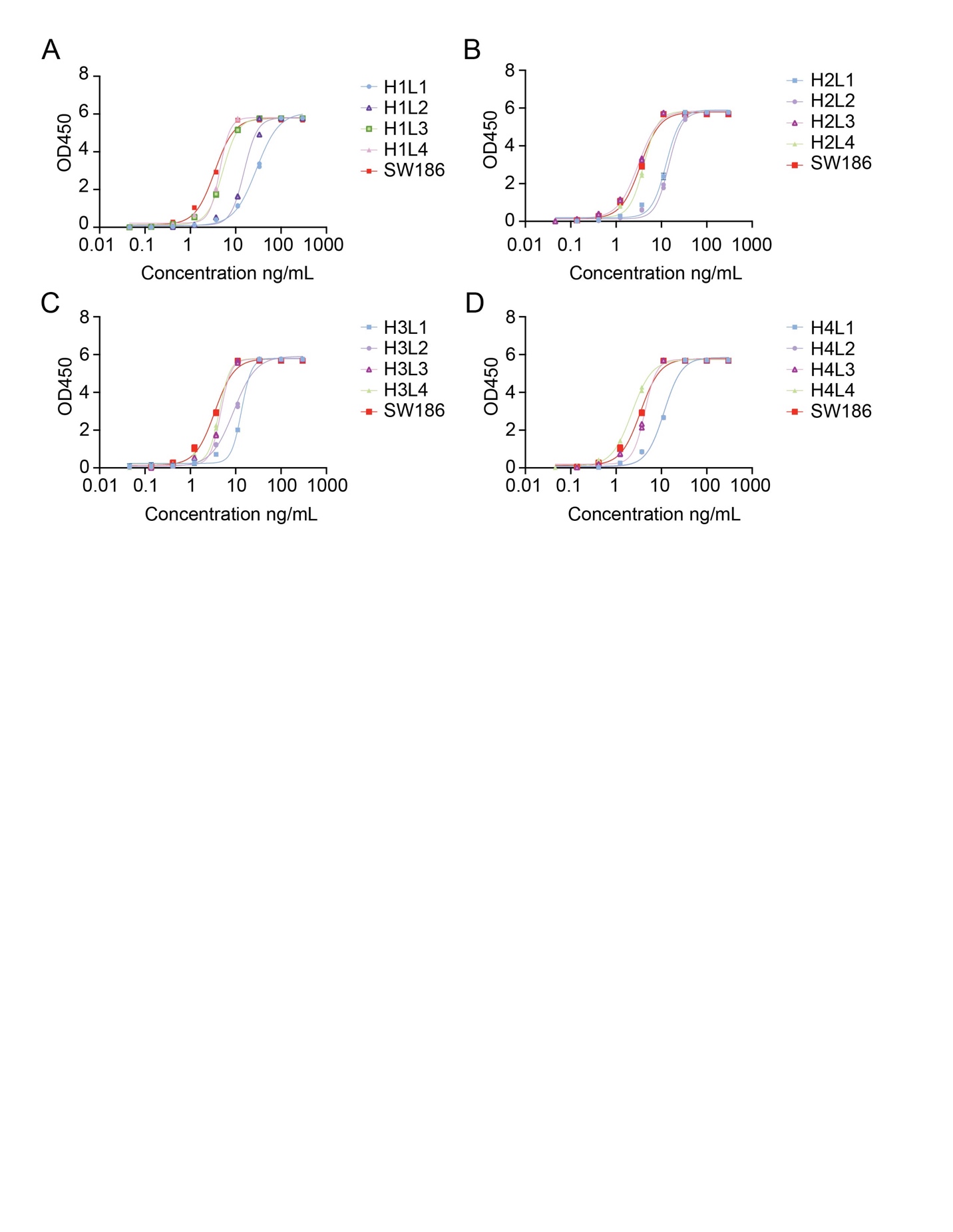


**Fig. S11. The binding affinity of humanized antibodies.** The combinations of four humanized heavy chains (H1-H4) and four humanized light chains (L1-L4) yielded 16 antibodies, which were expressed, purified and analyzed by ELISA using S-ecto as the coated antigen. Experiments were performed in duplicates. Each antibody dilution was done in duplicates.


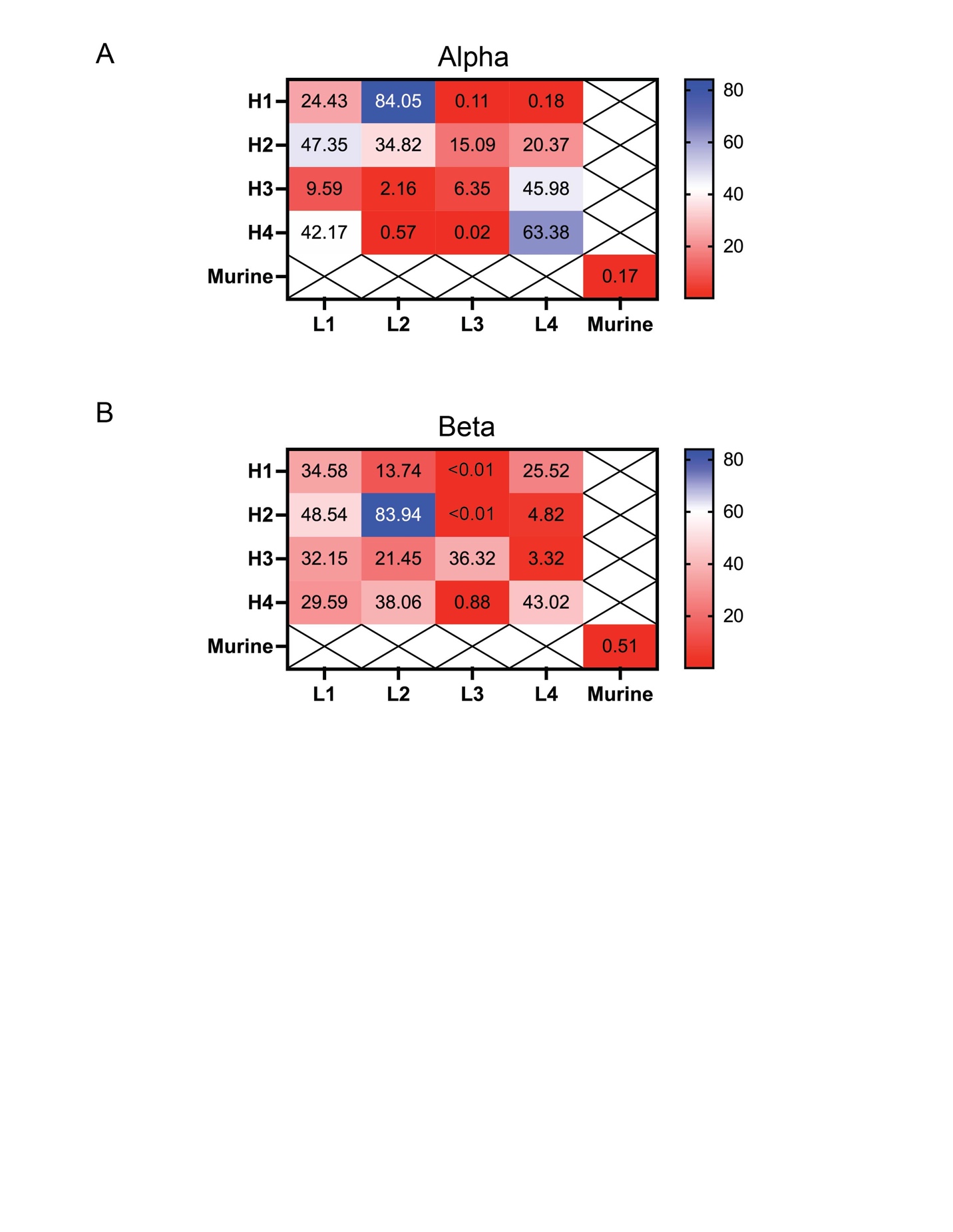


**Fig. S12. Neutralization activities of humanized antibodies against SARS-CoV-2 Alpha and Beta variants.** This plot summarized the neutralization IC_50_ (at ng/mL) of humanized SW186 antibodies against alpha (A) and beta pseudoviruses (B). Antibodies were serially diluted 1:3 from 30 μg/mL for 7 dilutions. Experiments were performed in duplicates for each antibody concentration.

**Table S1.**

Summary of isolated BCRs and their antigen specificity.

| Antigen | Qualified B cells | Distinct B cell clones | # of Ab tested (Freq>=3) | # of Ab that Binds Spike | # of Ab that Binds RBD |
| --- | --- | --- | --- | --- | --- |
| Spike | 268 | 146 | 18 | 13 | 6 |
| RBD | 265 | 131 | 21 | 21 | 21 |
| Total | 533 | 276 | 39 | 34 | 27 |

**Table S2. CryoEM Data Collection and Refinement Statistics**

|  | RBD/Fab |
| --- | --- |
| **Data collection and processing** |  |
| Magnification | 46,296 |
| Voltage (kV) | 300 |
| Electron exposure (e^–^/Å^2^) | 60 |
| Defocus range (μm) | 1.6 – 2.6 |
| Pixel size (Å) | 1.08 |
| Symmetry imposed | C1 |
| Initial particle images (no.) | 1,462,232 |
| Final particle images (no.) | 45,923 |
| Map resolution (Å) | 3.4 |
| FSC threshold | 0.143 |
|  |  |
| **Refinement** |  |
| Initial model used (PDB code) | 6XR8 |
| Model composition |  |
| Nonhydrogen atoms | 3840 |
| Protein residues | 486 |
| Ligands | NAG: 2  FUC: 1 |
| R.m.s. deviations |  |
| Bond lengths (Å) | 0.004 |
| Bond angles (°) | 0.639 |
|  |  |
| **Validation** |  |
| MolProbity score | 2.03 |
| Clashscore | 10.52 |
| Poor rotamers (%) | 0.00 |
| Ramachandran plot |  |
| Favored (%) | 91.84 |
| Allowed (%) | 8.16 |
| Disallowed (%) | 0.00 |

**Table S3. The amino acid sequences of humanized SW186.**

VH: heavy chain. VL: light chain. Underline labels CDR1-3. Back mutations were labeled by red color.

| Humanized Chain | Amino Acid sequences |
| --- | --- |
| SW186 heavy chain (murine) | QVQLQQSGAELVKPGASVKISCKAS**GVAFS**SYWMNWVKQRPGKGLEWIGQIYPGDGDTNYNGKFKGKATLTADKSSSTAYMQLSSLSSEDSAVYFCARGFIATVEETMDYWGQGTSVTVSS |
| VH1 | QVQLVQSGAEVKKPGSSVKVSCKASGVAFSSYWMNWVRQAPGQGLEWMGQIYPGDGDTNYNGKFKGRVTITADKSTSTAYMELSSLRSEDTAVYYCARGFIATVEETMDYWGQGTLVTVSS |
| VH2 | QVQLVQSGAEVKKPGSSVKVSCKASGVAFSSYWMNWVRQAPGQGLEWIGQIYPGDGDTNYNGKFKGRVTITADKSTSTAYMELSSLRSEDTAVYFCARGFIATVEETMDYWGQGTLVTVSS |
| VH3 | QVQLVQSGAEVKKPGSSVKVSCKASGVAFSSYWMNWVRQAPGQGLEWIGQIYPGDGDTNYNGKFKGRATITADKSTSTAYMELSSLRSEDTAVYFCARGFIATVEETMDYWGQGTLVTVSS |
| VH4 | QVQLVQSGAEVKKPGSSVKVSCKASGVAFSSYWMNWVKQAPGQGLEWIGQIYPGDGDTNYNGKFKGKATITADKSTSTAYMELSSLRSEDTAVYFCARGFIATVEETMDYWGQGTLVTVSS |
| SW186 light chain (murine) | DIQMTQTTSSLSASLGDRVTISCRASQDISNYLNWYQQKPDGTVKLLIYYTSRLHSGVPSRFSGSGSGTDYSLTISNLEQEDIATYFCQQSHTLPWTFGGGTKLEIK |
| VL1 | DIQMTQSPSSLSASVGDRVTITCRASQDISNYLNWYQQKPGKAPKLLIYYTSRLHSGVPSRFSGSGSGTDFTLTISSLQPEDFATYYCQQSHTLPWTFGGGTKLEIK |
| VL2 | DIQMTQSPSSLSASVGDRVTITCRASQDISNYLNWYQQKPGKAPKLLIYYTSRLHSGVPSRFSGSGSGTDFTLTISSLQPEDFATYFCQQSHTLPWTFGGGTKLEIK |
| VL3 | DIQMTQSPSSLSASVGDRVTITCRASQDISNYLNWYQQKPGKAPKLLIYYTSRLHSGVPSRFSGSGSGTDYTLTISSLQPEDFATYFCQQSHTLPWTFGGGTKLEIK |
| VL4 | DIQMTQSPSSLSASVGDRVTITCRASQDISNYLNWYQQKPGKTVKLLIYYTSRLHSGVPSRFSGSGSGTDYTLTISSLQPEDFATYFCQQSHTLPWTFGGGTKLEIK |

**Table S4. The binding kinetics and neutralization activity of humanized antibodies.**

| Antibody Name | Binding affinity against wildtype Spike protein | | | Neutralization against Delta Variant | |
| --- | --- | --- | --- | --- | --- |
|  | K_D_ (nM) ± sd | K_on_(Ms^-1^) | K_off_(s^-1^) | IC50 (ng/mL) | 95% confidence interval |
| SW186 | 1.09 ± 0.03 | 1.17 x10^5^ | 1.28 x10^-4^ | 9.82 | 7.32 – 13.07 |
| H1L1 | 10.01 ± 0.13 | 1.82 x10^5^ | 1.83 x10^-4^ | 32.02 | 14.83 – 77.2 |
| H1L2 | 26.16 ± 3.04 | 4.82 x10^4^ | 1.26 x10^-3^ | 19.68 | 11.66 – 34.03 |
| H1L3 | 4.37 ± 0.30 | 1.37 x10^5^ | 5.98 x10^-4^ | 13.88 | 6.19 – 32.75 |
| H1L4 | 5.03 ± 0.88 | 1.03 x10^5^ | 5.19 x10^-4^ | 12.81 | 7.99 – 20.50 |
| H2L1 | 18.07 ± 0.11 | 4.31 x10^4^ | 7.80 x10^-4^ | 695.70 | 245.50 – 1855.00 |
| H2L2 | 12.6 ± 0.30 | 1.20 x10^5^ | 1.51 x10^-3^ | 51.43 | 31.85 – 84.59 |
| H2L3 | 3.72 ± 0.07 | 1.91 x10^5^ | 7.13 x10^-4^ | 13.00 | 4.36 – 45.35 |
| H2L4 | 3.14 ± 0.02 | 1.92 x10^5^ | 6.03 x10^-4^ | 13.25 | 6.52 – 27.56 |
| H3L1 | 6.69 ± 0.08 | 1.78 x10^5^ | 1.19 x10^-3^ | 43.80 | 21.01 – 90.06 |
| H3L2 | 6.09 ± 0.07 | 2.01 x10^5^ | 1.22 x10^-3^ | 61.74 | 25.04 – 150.8 |
| H3L3 | 5.82 ± 0.07 | 1.31 x10^5^ | 7.61 x10^-4^ | 15.51 | 9.59 – 25.10 |
| H3L4 | 2.60 ± 0.02 | 1.81 x10^5^ | 4.69 x10^-4^ | 11.14 | 4.78 – 26.04 |
| H4L1 | 4.14 ± 0.03 | 1.86 x10^5^ | 7.70 x10^-4^ | 311.00 | 123.30 – 819.20 |
| H4L2 | 5.86 ± 0.04 | 1.63 x10^5^ | 9.53 x10^-4^ | 193.2 | 82.84 – 482.10 |
| H4L3 | 2.40 ± 0.02 | 1.77 x10^5^ | 4.24 x10^-4^ | 38.45 | 18.54 – 84.11 |
| H4L4 | 1.57 ± 0.01 | 2.16 x10^5^ | 3.38 x10^-4^ | 10.57 | 3.88 – 28.54 |
