# Supplementary material for: An antibody that neutralizes SARS-CoV-1 and SARS-CoV-2 by binding to a conserved spike epitope outside the receptor binding motif
Source: Sci Immunol. 2022 Aug 4:eabp9962. doi: 10.1126/sciimmunol.abp9962 (PMC9407945; doi:10.1126/sciimmunol.abp9962)
Supplement: Supplementary file 1 [file sciimmunol.abp9962_sm.pdf]

Science Immunology
AAAS

Supplementary Materials for

An antibody that neutralizes SARS-CoV-1 and SARS-CoV-2 by binding to a conserved spike epitope outside the receptor binding motif

Yan Fang *et al.*

Corresponding authors: Zhijian J. Chen, Zhijian.Chen@utsouthwestern.edu; Pei-Yong Shi, peshi@utmb.edu

DOI: 10.1126/sciimmunol.abp9962

The PDF file includes:

Figs. S1 to S12
Tables S1 to S4

Other Supplementary Material for this manuscript includes the following:

Table S5

An Antibody Neutralizes SARS-Cov-1 and SARS-Cov-2 by Binding To a Conserved Spike Epitope Outside the Receptor Binding Motif

Yan Fang[1, †], Pengcheng Sun[1, †], Xuping Xie[2,†], Mingjian Du[1], Fenghe Du[1], Jianfeng Ye[3], Birte K. Kalveram[2], Jessica A. Plante[5,6,7], Kenneth S. Plante[5,6,7], Bo Li[3], Xiao-chen Bai[4], Pei-Yong Shi[2,6,8*], Zhijian J. Chen[1,9,10,*]

[1] Department of Molecular Biology, University of Texas Southwestern Medical Center, Dallas, TX 75390-9148, USA

[2] Department of Biochemistry and Molecular Biology, University of Texas Medical Branch, 301 University Boulevard, Galveston, TX 77555-0144, USA

[3] Lyda Hill Department of Bioinformatics, University of Texas Southwestern Medical Center, Dallas, TX 75390, USA

[4] Department of Biophysics, University of Texas Southwestern Medical Center, Dallas, TX 75390, USA

[5]Department of Microbiology and Immunology, University of Texas Medical Branch, Galveston TX, USA

[6]Institute for Human Infections and Immunity, University of Texas Medical Branch, Galveston, TX, USA

[7]World Reference Center for Emerging Viruses and Arboviruses, University of Texas Medical Branch, Galveston, TX, USA

[8] Sealy Institute for Drug Discovery, University of Texas Medical Branch, Galveston, TX 77555, USA

[9] Center for Inflammation Research, University of Texas Southwestern Medical Center, Dallas, TX 75390-9148, USA

[10] Howard Hughes Medical Institute, University of Texas Southwestern Medical Center, Dallas, TX 75390-9148, USA

† These authors contributed equally

* Corresponding author. Email: Zhijian.Chen@utsouthwestern.edu or peshi@utmb.edu

List of supplementary materials

Fig. S1. The gating strategy of flow cytometry.
Fig. S2. The process of antibody filtering.
Fig. S3. The mutation chart of spike protein on pseudoviruses.
Fig. S4. Neutralization of pseudovirus displaying SARS-CoV-1 or WIV1 spike protein.
Fig. S5. Image processing of SW186 Fab-RBD-SD1 complex.
Fig. S6. Superposition of SW186 Fab-bound full length spike in different states.
Fig. S7. Structure-guided mutagenesis of SW186 and the effects on its binding to the spike protein.
Fig. S8. SW186 binds a conserved epitope on spike protein.
Fig. S9. Comparison between S309 and SW186 with key mutations on omicron.
Fig. S10. The neutralization activity of S309 against Omicron variants.
Fig. S11. The binding abilities of humanized antibodies.
Fig. S12. Neutralization activities of humanized antibodies against SARS-CoV-2 Alpha and Beta variants.

Table S1. Summary of isolated BCRs and their antigen specificity.
Table S2. CryoEM Data Collection and Refinement Statistics
Table S3. The amino acid sequences of humanized SW186.
Table S4. The binding kinetics and neutralization activity of humanized antibodies.
Table S5. Raw data and statistics used in this paper.

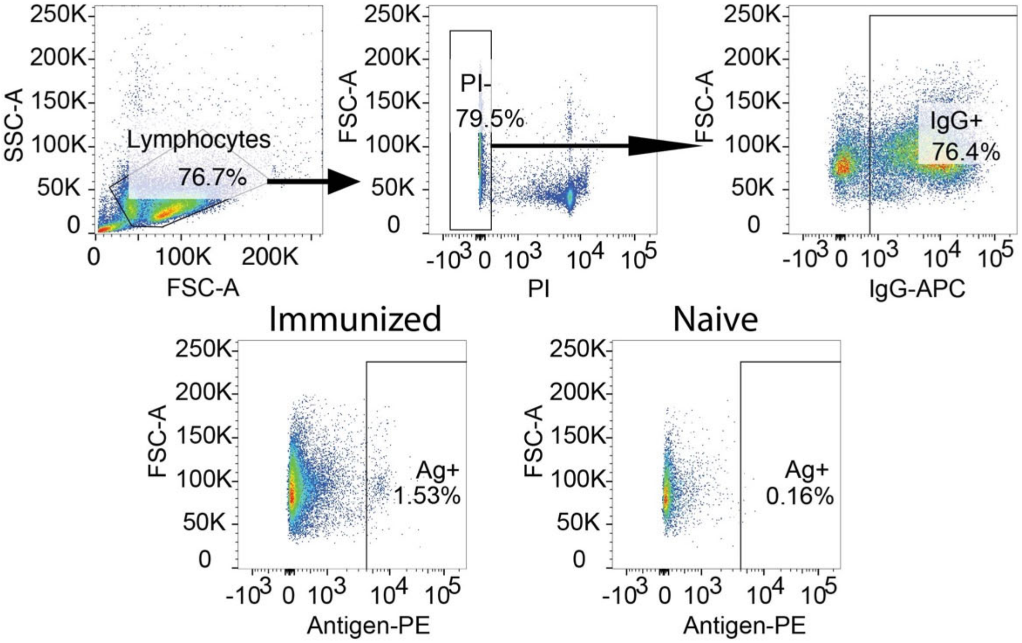

Fig. S1. The gating strategy of flow cytometry. In the PI- live cell gate, IgG-APC positive and PE-strep positive splenocytes (antigen positive) were sorted out. The splenocytes from naive (unimmunized) mice stained with the same regents show the background of this staining.

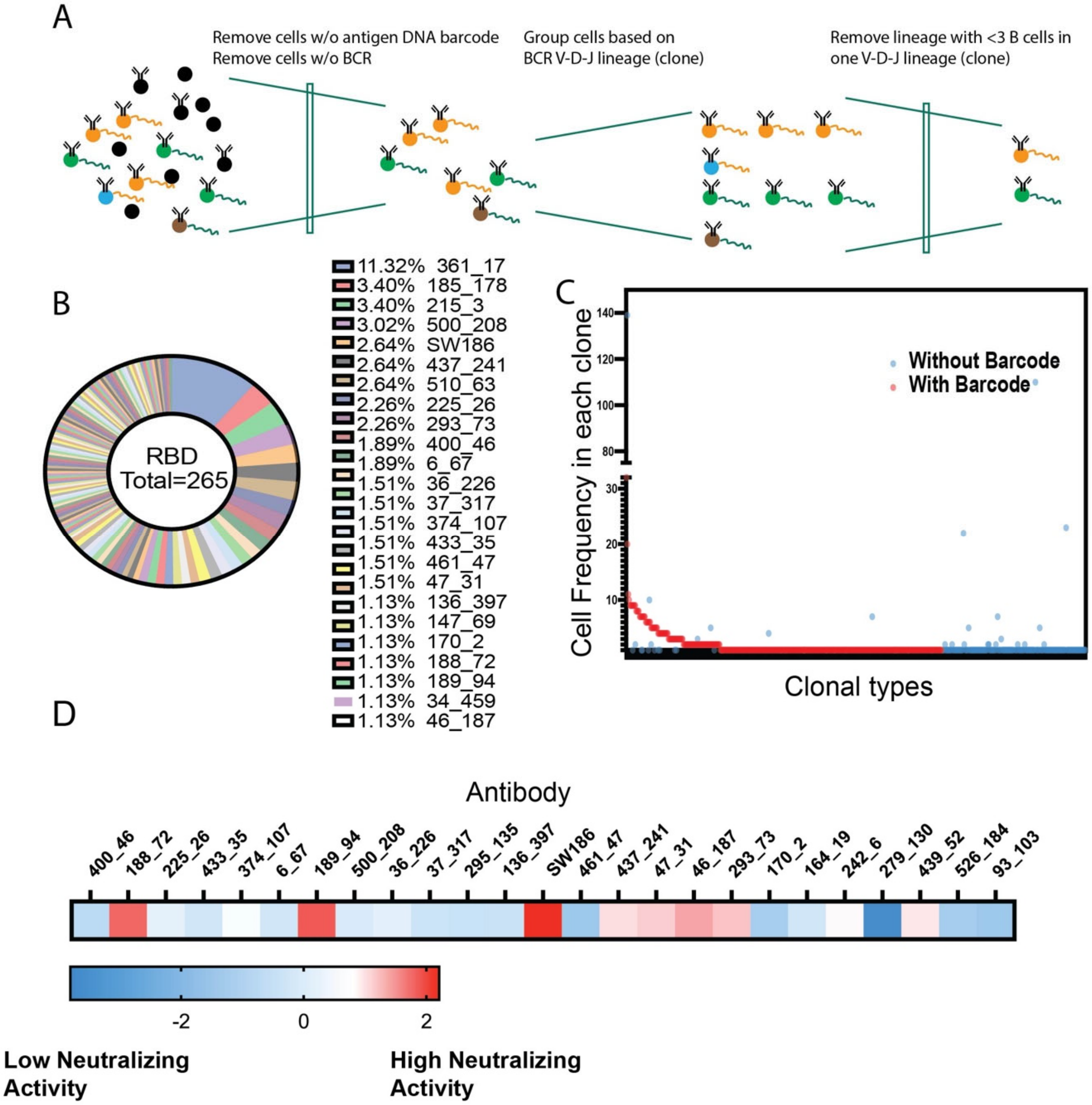

Fig. S2. The process of antibody filtering. (A) Illustration of antibody filtering during data analysis. The barcode negative or non-BCR cells were removed from the database. B cells were grouped into clones based on their germline sequences. Only B cells from expanded clones (B cells > 3 in each clone) are defined as antigen specific B cells and processed for further validation. (B) Clonal distribution of Spike-RBD specific BCRs. The percentage of expanded clones and their names are annotated on the right side of the figure. (C) The frequency of each B cell clone with (red) and without (blue) DNA barcodes. (D) The neutralizing activity (-log IC_{50} in ng/mL) of each antibody against the pseudovirus displaying the spike protein from the Wuhan-hu-1.

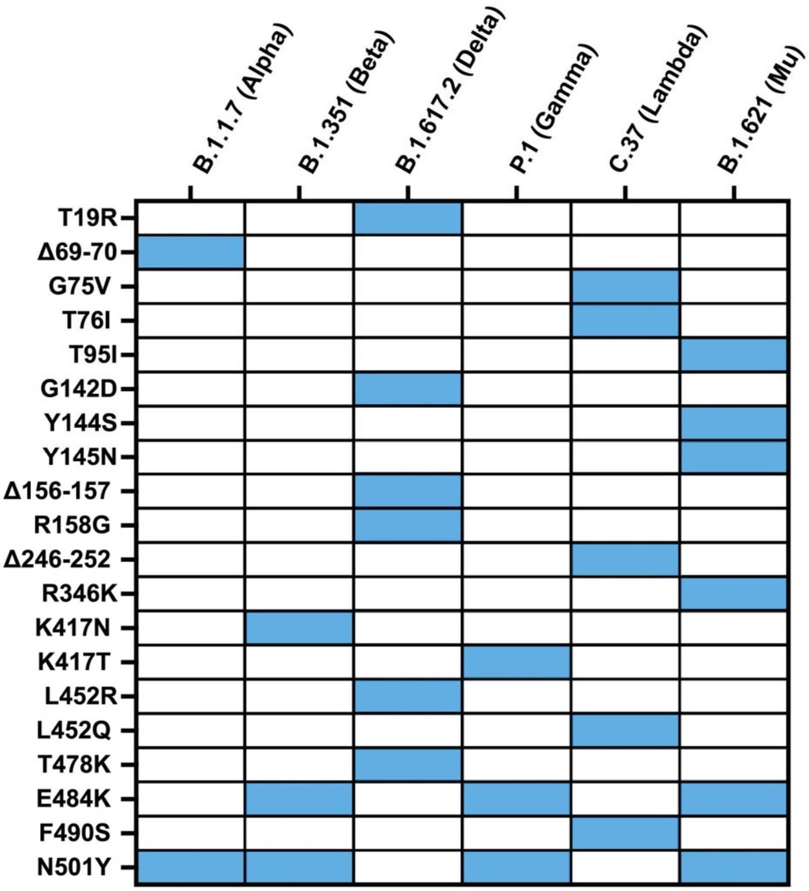

Fig. S3. The mutation chart of spike protein on pseudoviruses. To generate pseudoviruses variant to mimic SARS-CoV-2, we generated key mutations on spike proteins (labeled in blue) based on SARS-CoV-2 variant sequences.

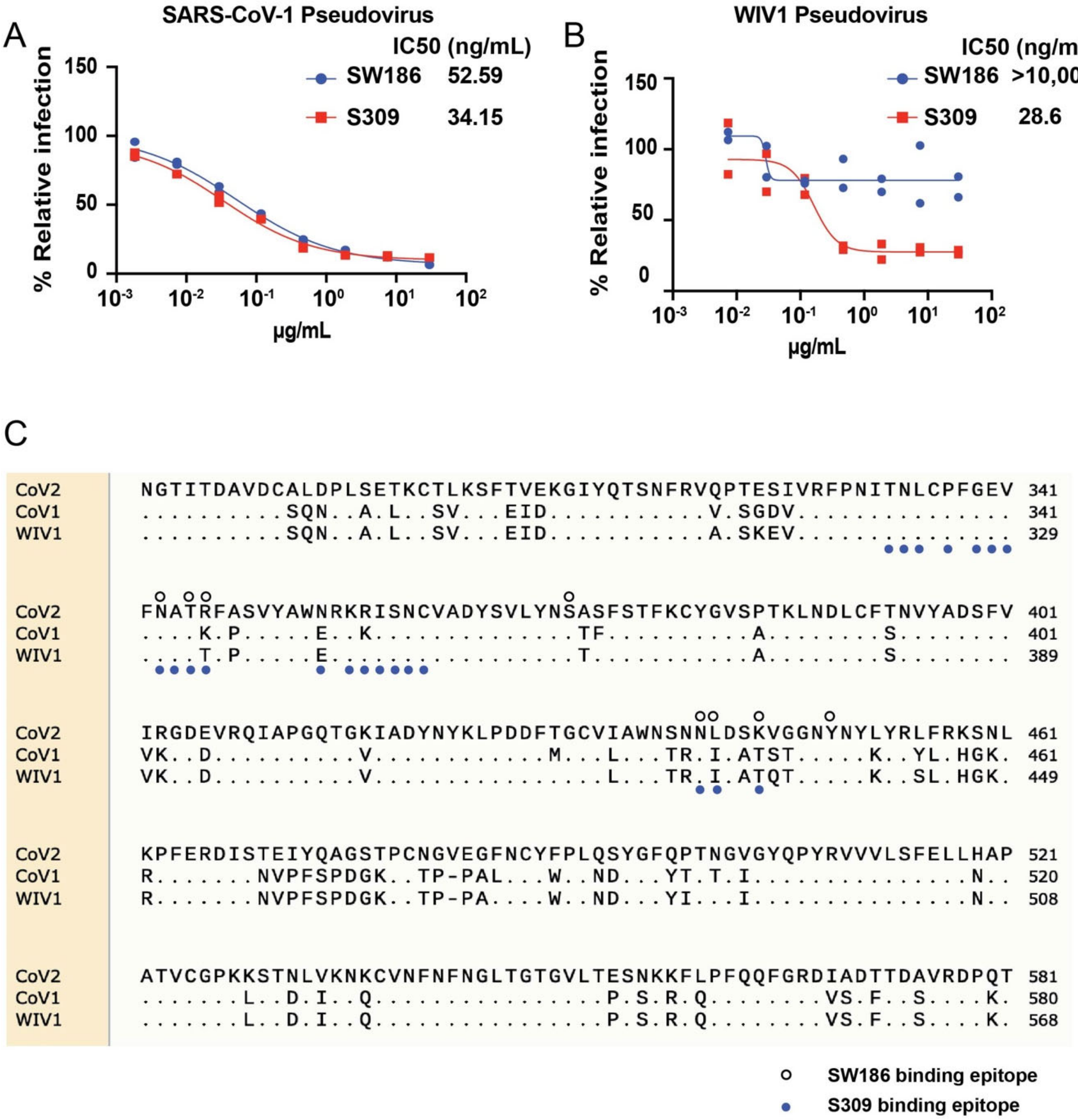

Fig. S4. Neutralization of pseudovirus displaying SARS-CoV-1 or WIV1 spike protein. Neutralization assays were performed using pseudotyped viruses displaying the SARA-CoV1 spike protein (A) or WIV1 spike protein (B). N=2. (C) The alignment of SARS-CoV-2, SARS-CoV-1, and WIV1 Spike proteins (focusing on RBD region). The binding epitopes of SW186 are indicated by black circles. The epitope of S309 are labeled based on literature (13).

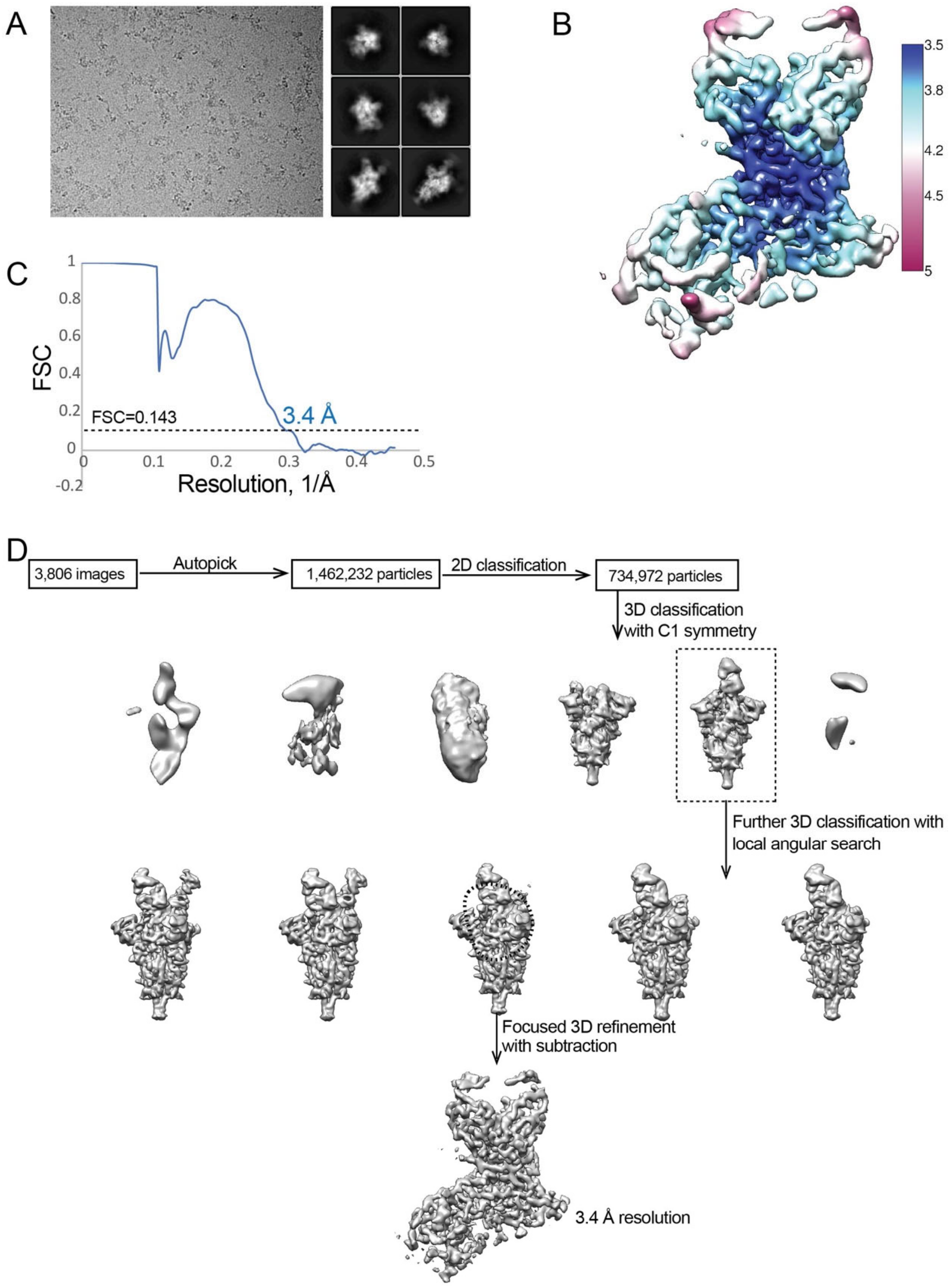

Fig. S5. Image processing of SW186 Fab-RBD-SD1 complex. (A) Cryo-EM micrograph (left) and 2D classification of SW186 Fab-spike. (B) Resolution map of locally refined Fab-RBD-SD1 map. (C) Gold-standard Fourier shell correlation curve for the local refinement. (D) Flowchart for image processing.

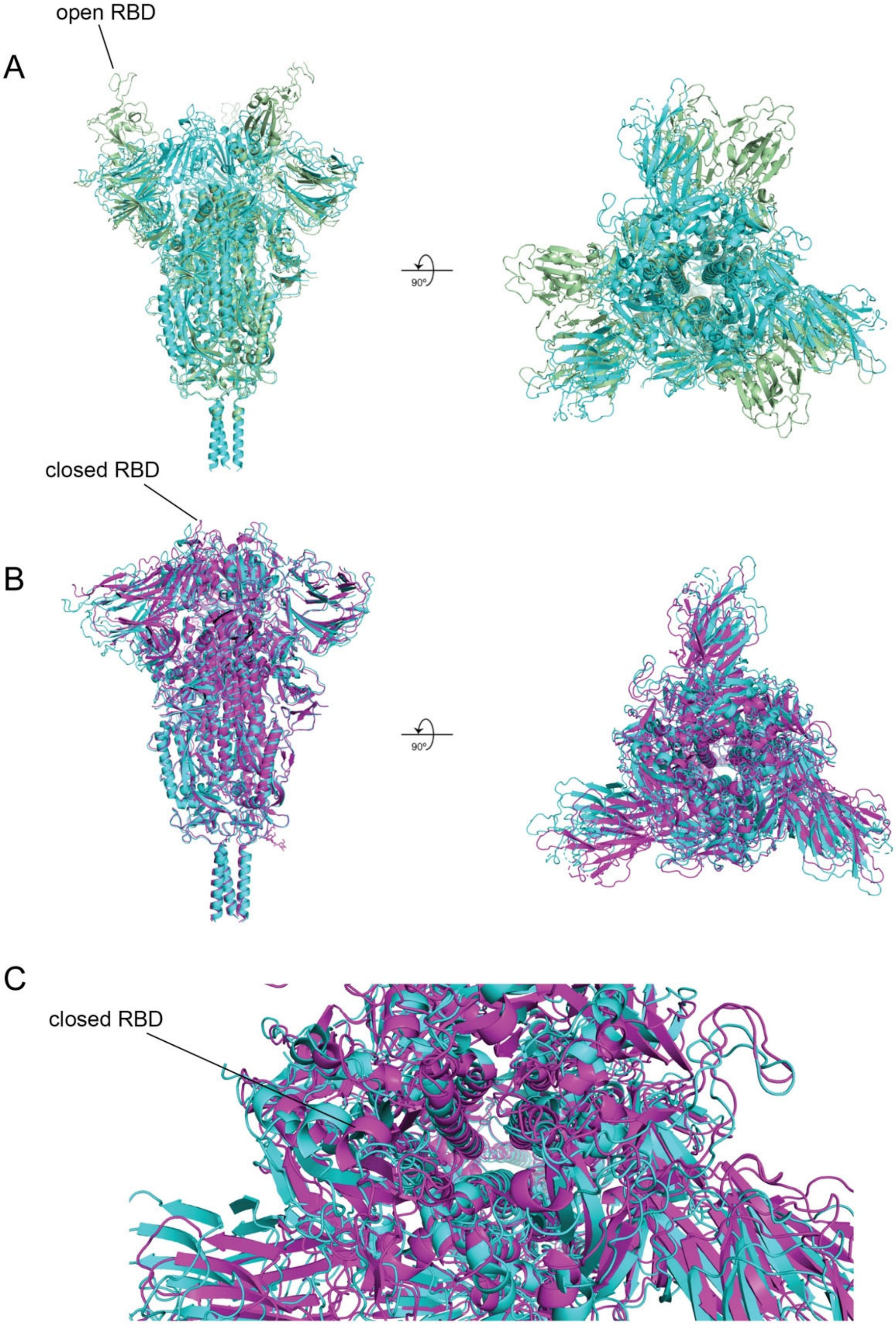

Fig. S6. Superposition of SW186 Fab-bound full length spike in different states. Both open (A; PDB code: 7CAK) and closed (B; PDB code: 6XR8) state models of spike are superimposed to SW186 Fab-bound Spike. Structure models are colored magenta, while the map is colored light grey. RBDs on the models are shown by lines. (C) Enlarged view of the close state model of spike shown in (B).

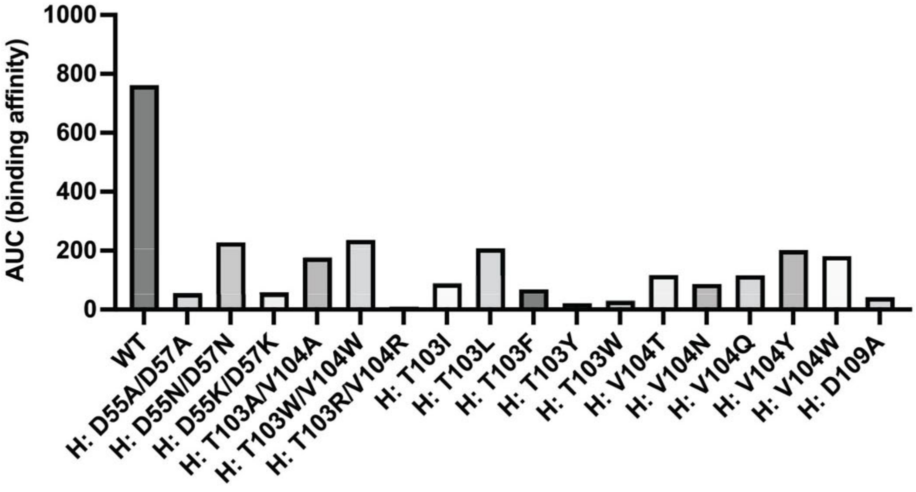

Fig. S7. Structure-guided mutagenesis of SW186 and the effects on its binding to the spike protein. The area under the curve (AUC) values were measured by ELISA assay. The unit of AUC is (μg/ml) * OD450.

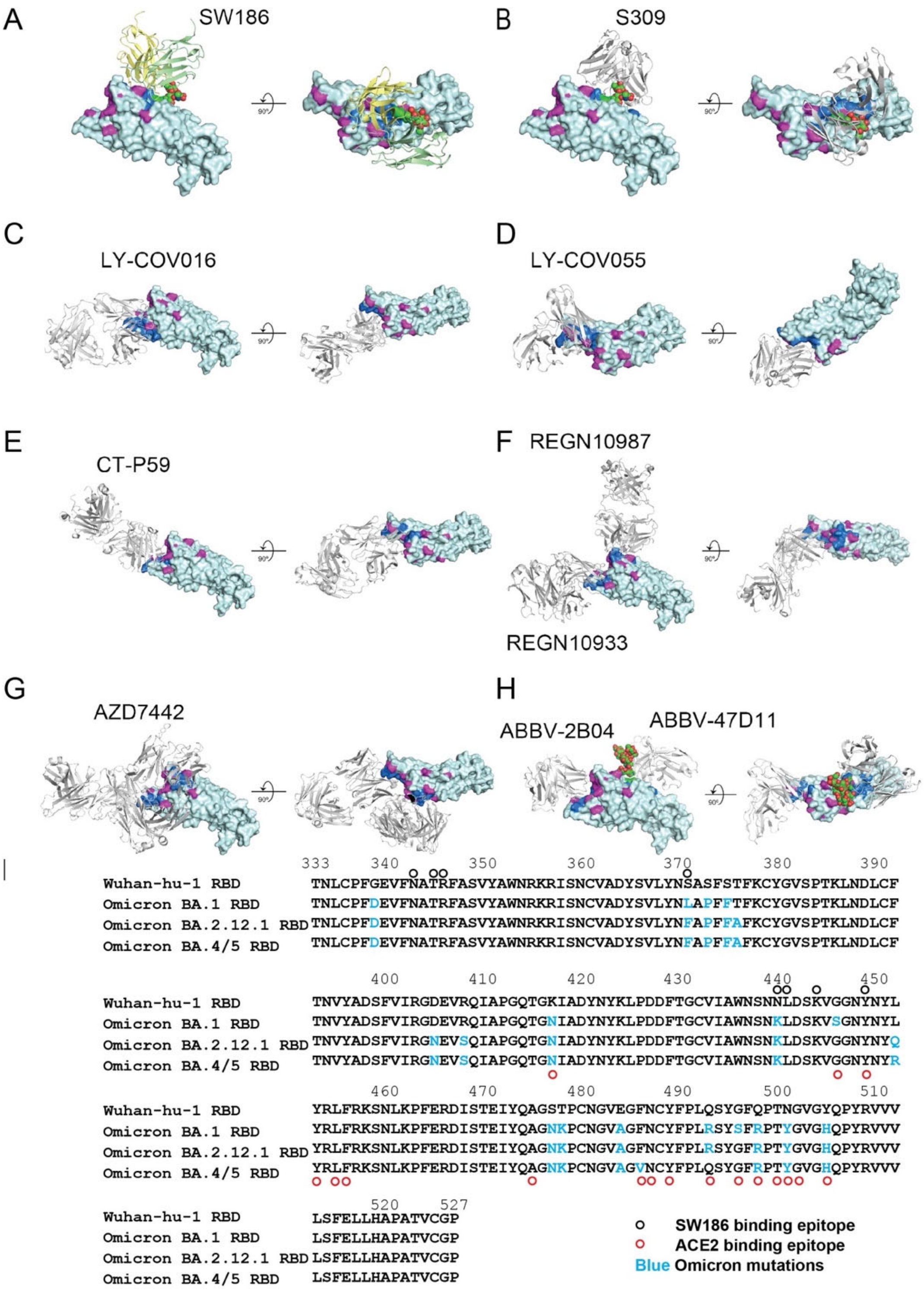

Fig. S8. SW186 binds a conserved epitope on spike protein. (A-H) Structural models of the Omicron RBD (pale cyan) bound to SW186 (A; V_H and V_L are colored pale yellow and pale green) and therapeutic antibodies (B-H; grey). Antibodies are shown as cartoon, and the RBD is presented as surface. The epitope of each antibody is colored marine, and the mutation sites of Omicron are colored magenta, including the overlap with the epitopes. N343-linked glycan is shown as green

spheres in A and B. PDB codes of the structures in these panels are: S309: 6WPS, LY-COV016: 7C01, LY-COV555: 7L3N, CT-P59: 7CM4, REGN mAbs: 6XDG, AZD7442: 7L7E, ABBV mAbs: 7AKJ, 7K9H. (I) Sequence alignment of RBDs from the Wuhan-hu-1 and Omicron subvariants of SARS-CoV-2. The mutation sites on Omicron are shown in light blue. The binding epitopes of SW186 and hACE2 are indicated by black and red circles, respectively.

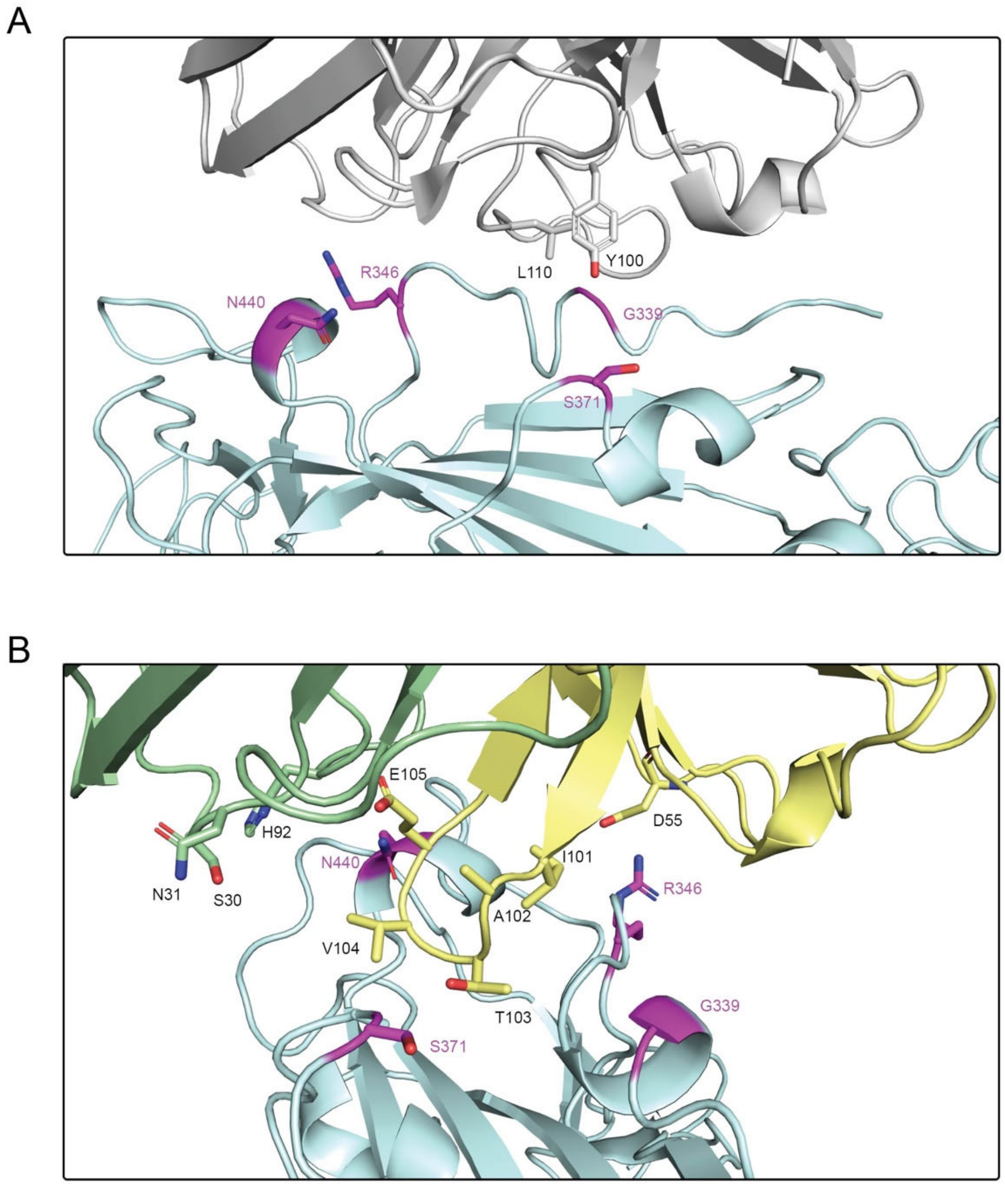

Fig. S9. Comparison between S309 and SW186 with key mutations on omicron. A zoom-in view of the antibodies S309 (A) and SW186 (B) binding to RBD. Mutations that largely decrease the neutralization activity of S309 are colored magenta. S309 and RBD are colored grey and pale cyan. V_H and V_L of SW186 are colored pale yellow and pale green. Surrounding residues are shown as sticks.

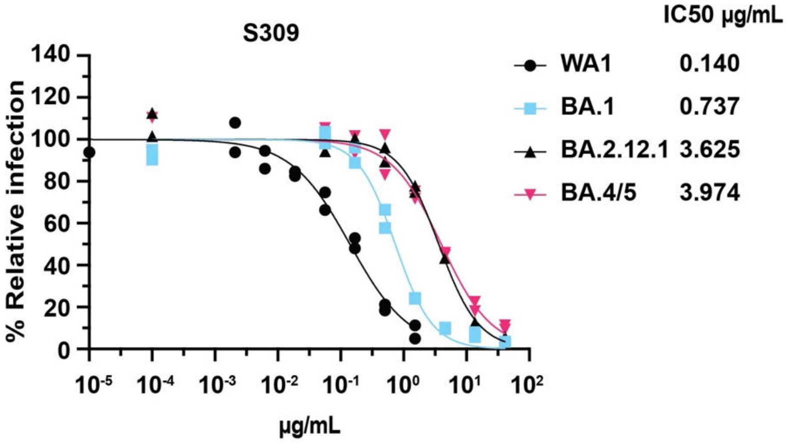

Fig. S10. The neutralization activity of S309 against Omicron variants. Neutralization assays were performed as in Figure 2 using recombinant SARS-CoV-2 omicron subvariants as indicated. Serial dilutions of the antibodies were done in duplicates. A four-parameter nonlinear regression was used to calculate the IC_{50}.

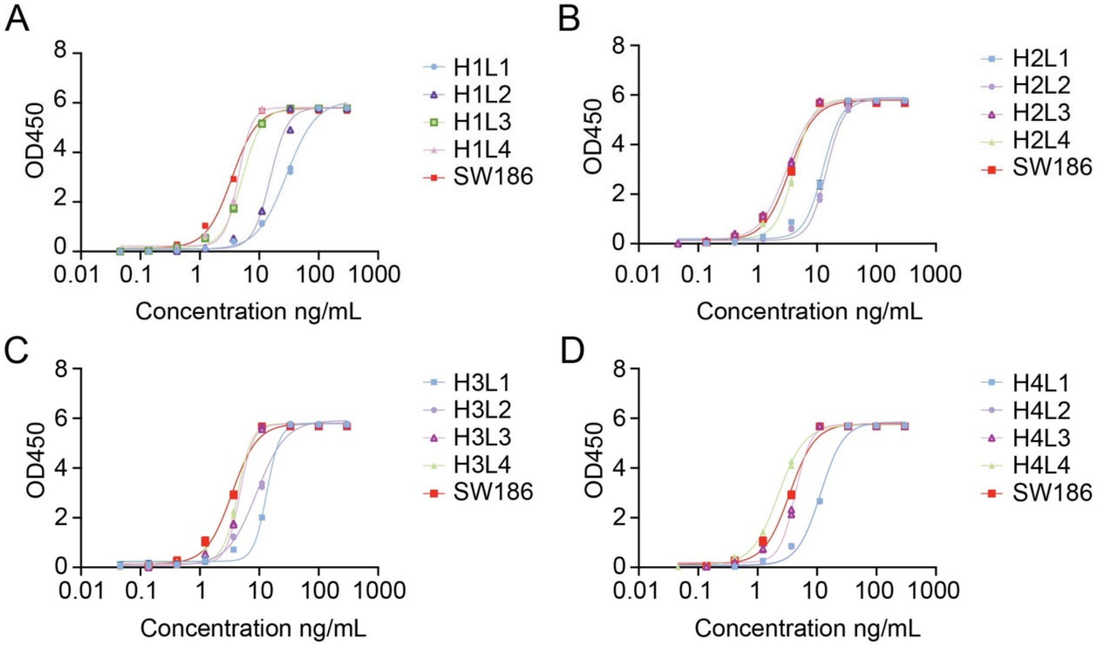

Fig. S11. The binding affinity of humanized antibodies. The combinations of four humanized heavy chains (H1-H4) and four humanized light chains (L1-L4) yielded 16 antibodies, which were expressed, purified and analyzed by ELISA using S-ecto as the coated antigen. Experiments were performed in duplicates. Each antibody dilution was done in duplicates.

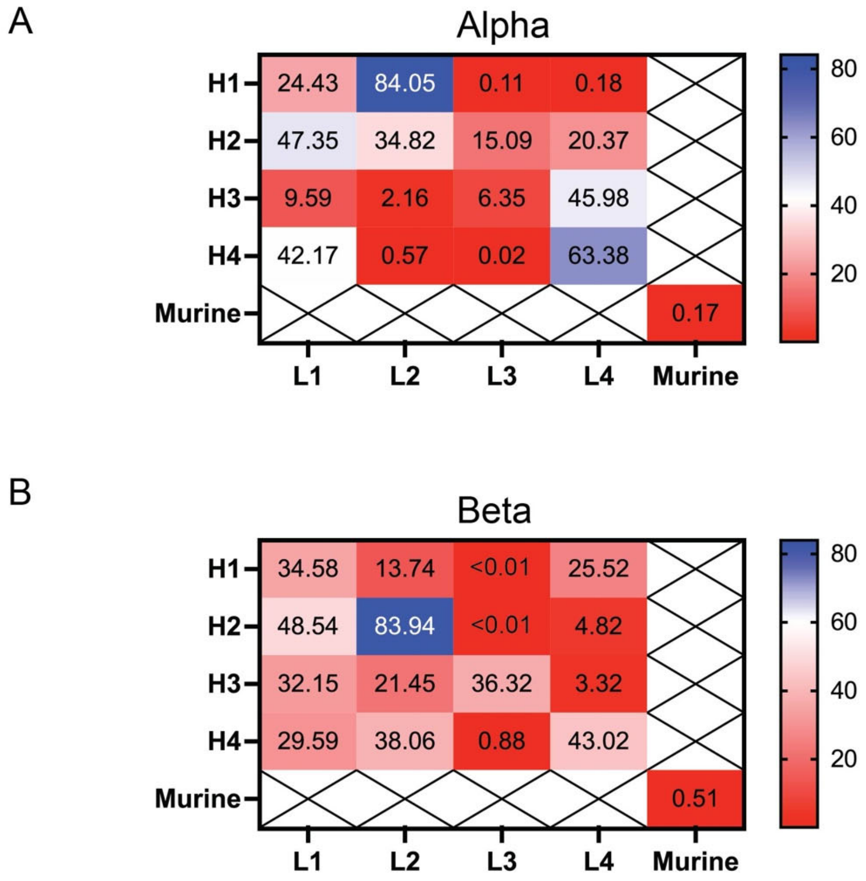

Fig. S12. Neutralization activities of humanized antibodies against SARS-CoV-2 Alpha and Beta variants. This plot summarized the neutralization IC_{50} (at ng/mL) of humanized SW186 antibodies against alpha (A) and beta pseudoviruses (B). Antibodies were serially diluted 1:3 from 30 μg/mL for 7 dilutions. Experiments were performed in duplicates for each antibody concentration.

Table S1.
Summary of isolated BCRs and their antigen specificity.

Antigen	Qualified B cells	Distinct B cell clones	# of Ab tested (Freq>=3)	# of Ab that Binds Spike	# of Ab that Binds RBD
Spike	268	146	18	13	6
RBD	265	131	21	21	21
Total	533	276	39	34	27

Table S2. CryoEM Data Collection and Refinement Statistics

	RBD/Fab
**Data collection and processing**	
Magnification	46,296
Voltage (kV)	300
Electron exposure (e^{-}/Å^2)	60
Defocus range (μm)	1.6 – 2.6
Pixel size (Å)	1.08
Symmetry imposed	C1
Initial particle images (no.)	1,462,232
Final particle images (no.)	45,923
Map resolution (Å)	3.4
FSC threshold	0.143
**Refinement**	
Initial model used (PDB code)	6XR8
Model composition	
Nonhydrogen atoms	3840
Protein residues	486
Ligands	NAG: 2<br>FUC: 1
R.m.s. deviations	
Bond lengths (Å)	0.004
Bond angles (°)	0.639
**Validation**	
MolProbity score	2.03
Clashscore	10.52
Poor rotamers (%)	0.00
Ramachandran plot	
Favored (%)	91.84
Allowed (%)	8.16
Disallowed (%)	0.00

Table S3. The amino acid sequences of humanized SW186.

VH: heavy chain. VL: light chain. Underline labels CDR1-3. Back mutations were labeled by red color.

Humanized Chain	Amino Acid sequences
SW186 heavy chain (murine)	QVQLQQSGAELVKPGASVKISCKAS**GVAFS**SYWMNWVKQRPGKGLEWIGQIYPGDGDTNYNGKFKGKATLTADKSSSTAYMQLSSLSSEDSAVYFCARGFIATVEETMDYWGQGTSVTVSS
VH1	QVQLVQSGAEVKKPGSSVKVSCKASGVAFSSYWMNWVRQAPGQGLEWMGQIYPGDGDTNYNGKFKGRVTITADKSTSTAYMELSSLRSEDTAVYYCARGFIATVEETMDYWGQGTLVTVSS
VH2	QVQLVQSGAEVKKPGSSVKVSCKASGVAFSSYWMNWVRQAPGQGLEWIGQIYPGDGDTNYNGKFKGRVTITADKSTSTAYMELSSLRSEDTAVYFCARGFIATVEETMDYWGQGTLVTVSS
VH3	QVQLVQSGAEVKKPGSSVKVSCKASGVAFSSYWMNWVRQAPGQGLEWIGQIYPGDGDTNYNGKFKGRATITADKSTSTAYMELSSLRSEDTAVYFCARGFIATVEETMDYWGQGTLVTVSS
VH4	QVQLVQSGAEVKKPGSSVKVSCKASGVAFSSYWMNWVKQAPGQGLEWIGQIYPGDGDTNYNGKFKGKATITADKSTSTAYMELSSLRSEDTAVYFCARGFIATVEETMDYWGQGTLVTVSS
SW186 light chain (murine)	DIQMTQTTSSLSASLGDRVTISCRASQDISNYLNWYQQKPDGTVKLLIYYTSRLHSGVPSRFSGSGSGTDYSLTISNLEQEDIATYFCQQSHTLPWTFGGGTKLEIK
VL1	DIQMTQSPSSLSASVGDRVTITCRASQDISNYLNWYQQKPGKAPKLLIYYTSRLHSGVPSRFSGSGSGTDFTLTISSLQPEDFATYYCQQSHTLPWTFGGGTKLEIK
VL2	DIQMTQSPSSLSASVGDRVTITCRASQDISNYLNWYQQKPGKAPKLLIYYTSRLHSGVPSRFSGSGSGTDFTLTISSLQPEDFATYFCQQSHTLPWTFGGGTKLEIK
VL3	DIQMTQSPSSLSASVGDRVTITCRASQDISNYLNWYQQKPGKAPKLLIYYTSRLHSGVPSRFSGSGSGTDYTLTISSLQPEDFATYFCQQSHTLPWTFGGGTKLEIK
VL4	DIQMTQSPSSLSASVGDRVTITCRASQDISNYLNWYQQKPGKTVKLLIYYTSRLHSGVPSRFSGSGSGTDYTLTISSLQPEDFATYFCQQSHTLPWTFGGGTKLEIK

Table S4. The binding kinetics and neutralization activity of humanized antibodies.

Antibody Name	Binding affinity against wildtype Spike protein			Neutralization against Delta Variant	
	K_D (nM) ± sd	$K_{on}(Ms^{-1})$	$K_{off}(s^{-1})$	IC50 (ng/mL)	95% confidence interval
SW186	1.09 ± 0.03	1.17 x10^5	1.28 x10^{-4}	9.82	7.32 – 13.07
H1L1	10.01 ± 0.13	1.82 x10^5	1.83 x10^{-4}	32.02	14.83 – 77.2
H1L2	26.16 ± 3.04	4.82 x10^4	1.26 x10^{-3}	19.68	11.66 – 34.03
H1L3	4.37 ± 0.30	1.37 x10^5	5.98 x10^{-4}	13.88	6.19 – 32.75
H1L4	5.03 ± 0.88	1.03 x10^5	5.19 x10^{-4}	12.81	7.99 – 20.50
H2L1	18.07 ± 0.11	4.31 x10^4	7.80 x10^{-4}	695.70	245.50 – 1855.00
H2L2	12.6 ± 0.30	1.20 x10^5	1.51 x10^{-3}	51.43	31.85 – 84.59
H2L3	3.72 ± 0.07	1.91 x10^5	7.13 x10^{-4}	13.00	4.36 – 45.35
H2L4	3.14 ± 0.02	1.92 x10^5	6.03 x10^{-4}	13.25	6.52 – 27.56
H3L1	6.69 ± 0.08	1.78 x10^5	1.19 x10^{-3}	43.80	21.01 – 90.06
H3L2	6.09 ± 0.07	2.01 x10^5	1.22 x10^{-3}	61.74	25.04 – 150.8
H3L3	5.82 ± 0.07	1.31 x10^5	7.61 x10^{-4}	15.51	9.59 – 25.10
H3L4	2.60 ± 0.02	1.81 x10^5	4.69 x10^{-4}	11.14	4.78 – 26.04
H4L1	4.14 ± 0.03	1.86 x10^5	7.70 x10^{-4}	311.00	123.30 – 819.20
H4L2	5.86 ± 0.04	1.63 x10^5	9.53 x10^{-4}	193.2	82.84 – 482.10
H4L3	2.40 ± 0.02	1.77 x10^5	4.24 x10^{-4}	38.45	18.54 – 84.11
H4L4	1.57 ± 0.01	2.16 x10^5	3.38 x10^{-4}	10.57	3.88 – 28.54
